# Single-cell transcriptomics analysis of proliferative diabetic retinopathy fibrovascular membranes reveals AEBP1 as fibrogenesis modulator

**DOI:** 10.1172/jci.insight.172062

**Published:** 2023-11-02

**Authors:** Katia Corano Scheri, Jeremy A. Lavine, Thomas Tedeschi, Benjamin R. Thomson, Amani A. Fawzi

**Affiliations:** 1 Department of Ophthalmology and; 2 Cardiovascular and Renal Research Institute, Center for Kidney Research and Therapeutics, Feinberg School of Medicine, Northwestern University, Chicago, Illinois, USA.

**Keywords:** Angiogenesis, Ophthalmology, Diabetes, Fibrosis, Pericytes

## Abstract

The management of preretinal fibrovascular membranes, a devastating complication of advanced diabetic retinopathy (DR), remains challenging. We characterized the molecular profile of cell populations in these fibrovascular membranes to identify potentially new therapeutic targets. Preretinal fibrovascular membranes were surgically removed from patients and submitted for single-cell RNA-Seq (scRNA-Seq). Differential gene expression was implemented to define the transcriptomics profile of these cells and revealed the presence of endothelial, inflammatory, and stromal cells. Endothelial cell reclustering identified subclusters characterized by noncanonical transcriptomics profile and active angiogenesis. Deeper investigation of the inflammatory cells showed a subcluster of macrophages expressing proangiogenic cytokines, presumably contributing to angiogenesis. The stromal cell cluster included a pericyte-myofibroblast transdifferentiating subcluster, indicating the involvement of pericytes in fibrogenesis. Differentially expressed gene analysis showed that Adipocyte Enhancer-binding Protein 1, *AEBP1*, was significantly upregulated in myofibroblast clusters, suggesting that this molecule may have a role in transformation. Cell culture experiments with human retinal pericytes (HRP) in high-glucose condition confirmed the molecular transformation of pericytes toward myofibroblastic lineage. *AEBP1* siRNA transfection in HRP reduced the expression of profibrotic markers in high glucose. In conclusion, AEBP1 signaling modulates pericyte-myofibroblast transformation, suggesting that targeting AEBP1 could prevent scar tissue formation in advanced DR.

## Graphical abstract

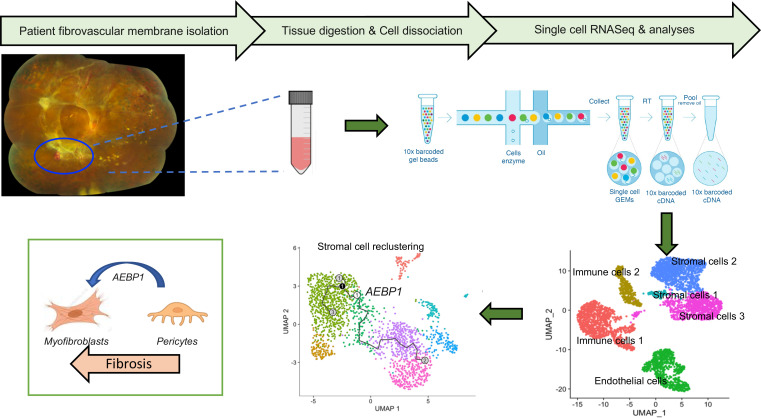

## Introduction

Diabetic retinopathy (DR) is a serious complication of diabetes mellitus and the most severe complication on an individual patient’s quality of life (1, 2). Based on the level of microvascular degeneration and related ischemic damage, DR progresses from nonproliferative stages (NPDR) to advanced, proliferative DR (PDR). With progressive ischemia, progression to PDR is associated with preretinal proliferation of disorganized angiogenesis, which ultimately leads to preretinal fibrovascular membranes and tractional retinal detachments, with devastating visual consequences unless promptly treated with surgery (3–5). Current pharmacologic treatments address vascular leakage and angiogenesis with intravitreal injections of anti–vascular endothelial growth factor (anti-VEGF) or corticosteroids, though a subgroup of patients do not respond to these treatments (6). Despite the availability of other options — including laser therapy or eye surgery, such as vitrectomy for extensive bleeding or retinal detachments due to fibrovascular scar tissue formation (7, 8) — there is an unmet need for therapies that prevent fibrovascular proliferation in the eye, the main driver of the end stages of DR.

The retinal neurovascular unit refers to the interdependency between vascular endothelial cells, pericytes, glia, neurons, and immune cells. A better understanding of the contribution of each cell type to DR progression and the molecular mechanisms regulating their crosstalk could be the key to identify new therapeutic targets. In addition to retinal vascular alterations, fibrovascular scarring can ultimately lead to blindness (9). Fibrosis is an intricate, reparative process that develops in response to acute or chronic injury. The mechanisms that drive fibrosis in DR are not fully understood; hence, there is a great need to better understand this important cause of vision loss. This would be a first step toward new therapies to address progressive preretinal fibrosis and traction detachment and to avoid the concomitant poor visual and anatomic outcomes.

In this study, we focused on the fibrovascular stage by studying 4 membranes surgically extracted from patients who required surgery for complications of PDR. We performed single-cell RNA-Seq (scRNA-Seq), which identified the main cell populations in these membranes as well as in endothelial, immune, and stromal cells. We then investigated the molecular profile of each population and found that dysfunctional endothelial cells and an angiogenic cluster of macrophages actively contributed to the angiogenetic process. We also identified a molecule that modulates the transition of pericytes toward a myofibroblastic phenotype and confirmed its role using in vitro studies. Our data shed light on a potential therapeutic target that can be harnessed to address preretinal fibrosis and ameliorate sight-threatening complications of DR.

## Results

### Cell cluster identification.

**Figure 1:**
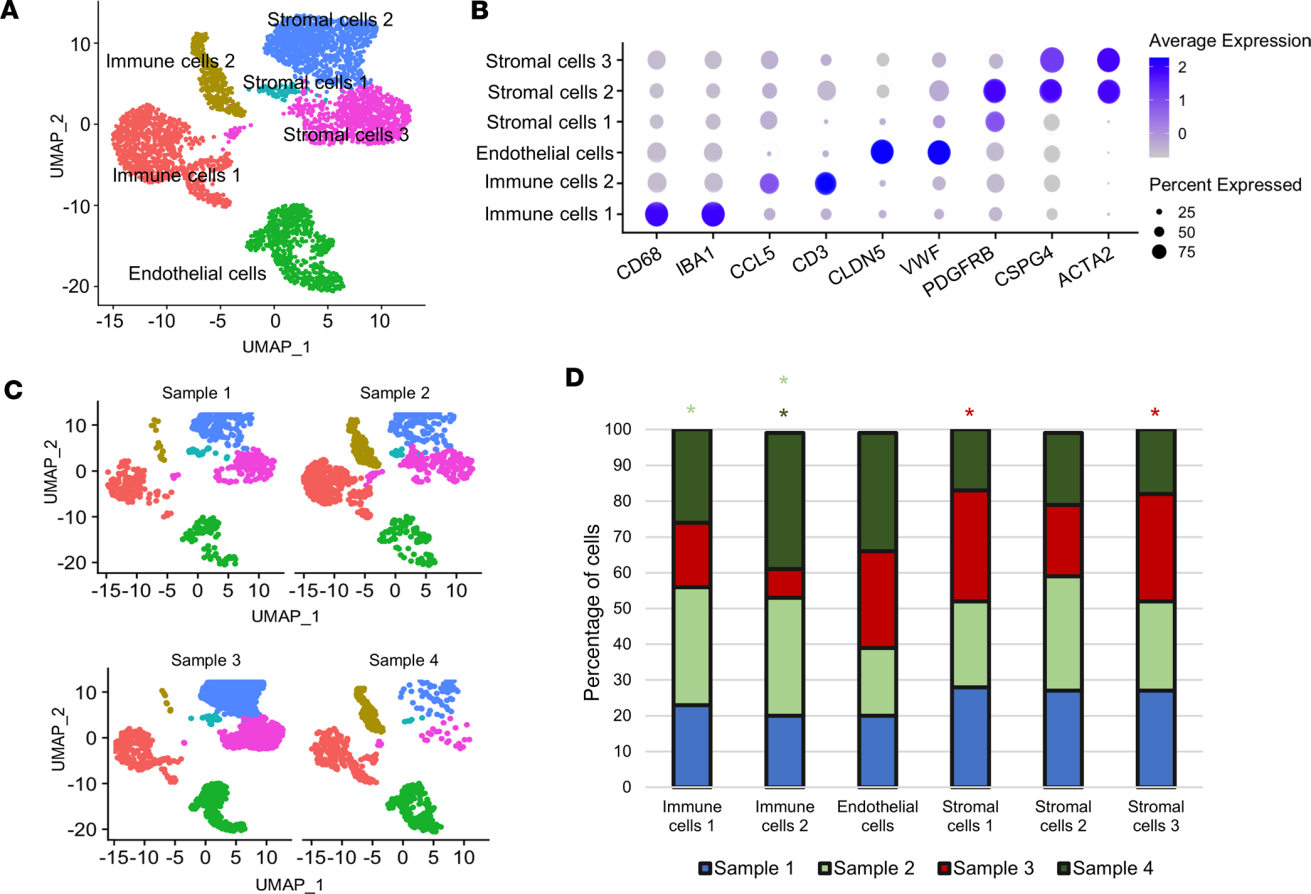
scRNA-Seq analyses and cluster annotation. (**A**) Representative Uniform Manifold Approximation and Projection (UMAP) plot of the 6 different clusters revealed by Seurat analysis conducted in R Studio, and cluster identification. (**B**) Dot plot for common cell-specific markers, such as *CD68*, *IBA1*, *CCL5*, *CD3*, *CLDN5*, *VWF*, *PDGFRB*, *CSPG4*, and *ACTA2*. Cell type classification and the percentage and average expression of the specific cell markers are shown. (**C** and **D**) UMAP plot split by sample and the proportion of cells derived from each samples. Color of asterisk in **D** identifies which groups were enriched by hypergeometric enrichment test (*P* < 0.001).

Human fibrovascular membranes from patients with PDR were collected to profile the cell populations. The membranes were surgically removed and, after tissue digestion to obtain single-cell suspension, were submitted to scRNA-Seq. Raw data from 4 samples were individually processed for quality check and doublet removal (Supplemental Figure 1; supplemental material available online with this article; https://doi.org/10.1172/jci.insight.172062DS1) and were then integrated in a single data set for downstream analyses. As reported in the Uniform Manifold Approximation and Projection (UMAP), we identified 6 different clusters (Figure 1A). Using canonical markers of established cell types, we identified that immune cells 1 included macrophages, expressing *CD68* and *IBA1*, and immune cells 2 expressed high levels of *CCL5* and *CD3*, consistent with T cells. The endothelial cells were identified based on the canonical endothelial marker *CLDN5* and *VWF*, and the stromal cells were identified using the markers *PDGFRβ*, *CSPG4*, and *ACTA2*. The dot plot in Figure 1B shows the cell type classification, the percentage and average expression of specific cell markers. The cell clustering of each sample and the proportional contribution from the 4 samples to each cluster are shown in the UMAP in Figure 1C and in the bar plot in Figure 1D, respectively. Hypergeometric distribution analysis showed that sample 2 was enriched for macrophages and T cells, sample 3 was enriched for stromal cells 1 and 3, and sample 4 was enriched for T cells (Figure 1D; *P* < 0.001). Interestingly, endothelial cells were evenly distributed among the samples. To better investigate the molecular profile of the identified cells, we next reclustered each cell type separately.

### Abnormal endothelial cells drive angiogenesis in PDR fibrovascular membranes.

**Figure 2:**
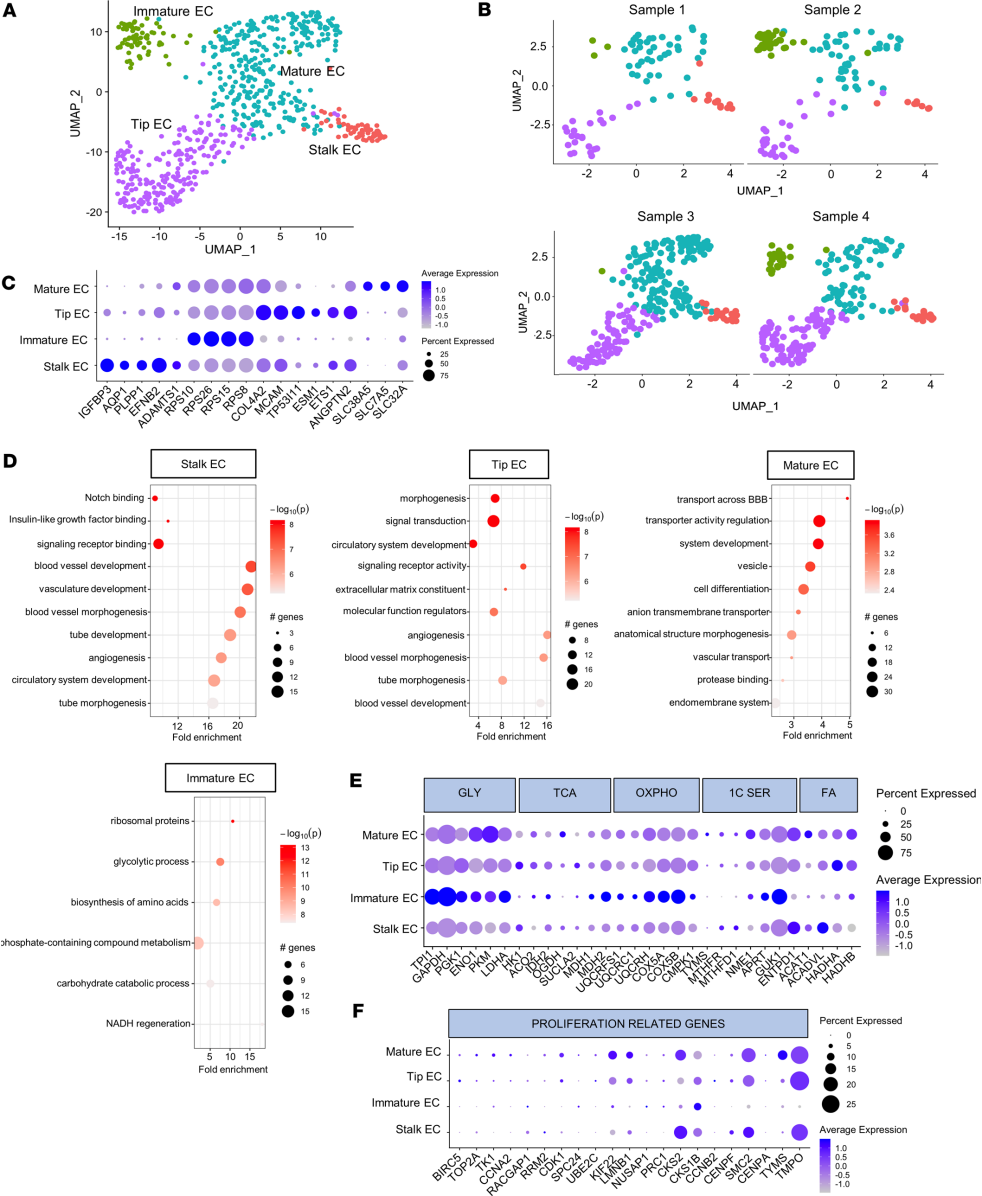
Endothelial cell clustering, classification, and pathway enrichment analysis. (**A** and **B**) Representative UMAP plot of the 4 different cluster of endothelial cells is shown in **A**, while UMAP plot for each sample is shown in **B**. (**C**) Dot plot for the most common enriched genes for immature, stalk, tip, and mature endothelial cells. Cell type classification and the percentage and average expression of the specific cell markers are shown. (**D**) Dot plot of the pathway enrichment analysis for the upregulated genes of each cluster using gProfiler and PathFindR package. Only the most significant differentially expressed genes (log_2_FC > 1 and *P*_adj_ < 0.01) were chosen for pathway enrichment analysis. The graph shows the number of genes modulated in each single pathway, the fold enrichment, and the statistical significance. (**E**) Dot plot showing the metabolic pathway gene expression. (**F**) Dot plot showing a panel of proliferation-related genes.

The endothelial cells were subclustered into 4 different groups using well-known enriched genes (10–12) (Figure 2A). Their relative distribution in each sample is shown in Figure 2B. The angiogenetic process is usually initiated by specialized endothelial cells, the tip cells, that sense the microenvironment, emit filopodia, and start the sprouting process, identified with the gene expression of *COL4A2*, *MCAM*, *TP53I11*, *ESM1*, and *ANGPTN2* (Figure 2C). Cells expressing *IGFBP3*, *AQP1*, *PLPP1*, *EFNB2*, and *ADAMTS1* were classified as stalk cells, the cells responsible for proliferating and elongating the neovascular sprout during angiogenesis. Mature endothelial cells, responsible for the most specialized functions, were identified with the expression of enriched genes such as *SLC38A5*, *SLC7A5*, and *SLC32A*. Immature endothelial cells were identified using the enriched genes *RPS10*, *RPS26*, *RPS15*, and *RPS8* (Figure 2C). Immature endothelial cells are generally characterized by upregulated ribosomal gene expression and the absence of any specific endothelial-type gene expression, consistent with an intermediate phenotype (13).

Pathway enrichment analysis for the upregulated genes was performed on the most significantly expressed genes (log_2_ fold change [log_2_FC > 1], adjusted *P* value [*P*_adj_ < 0.01]). As expected, pathway-enriched genes were primarily related to a processes related to active angiogenesis in stalk and tip endothelial cells, such as blood vessel development, vasculature development, and angiogenesis as shown in the dot plots in Figure 2D. In addition, the Notch pathway, a well-known modulator of stalk cell physiology, was among the modulated pathways in this cluster. Pathways such as transporter activity regulation, vesicles, and transport across blood-brain barrier, are shown as enriched in mature endothelial cells, as expected. Immature cells showed enriched pathways related to ribosomal proteins, as expected, and glycolysis (Figure 2D). The latter pathway led us to evaluate the metabolic gene panel in all the endothelial cells. We detected a differential expression of glycolytic molecules in all the endothelial cell sublusters, a generally higher expression of these molecules compared with the tricarboxylic acid cycle genes, and a very similar expression level if compared with oxidative phosphorylation (OXPHOS) and nucleotide biosynthesis genes (Figure 2E). We observed that the metabolism was highly active in the immature endothelial cells, confirming the enrichment pathway analysis results. We also explored proliferation markers and found that they had a similar expression level among all the clusters, with the exception of immature cells, which showed low level of expression of proliferation-related genes (Figure 2F).

Pathway enrichment analysis for the downregulated genes (log_2_FC < –1, *P*_adj_ < 0.01) is reported in the Supplemental Figure 2. As expected, genes related to angiogenesis and blood vessel development were downregulated in immature and mature endothelial cells. On the other hand, gap junction– and transporter activity–related genes were downregulated in the stalk and tip cells.

**Figure 3:**
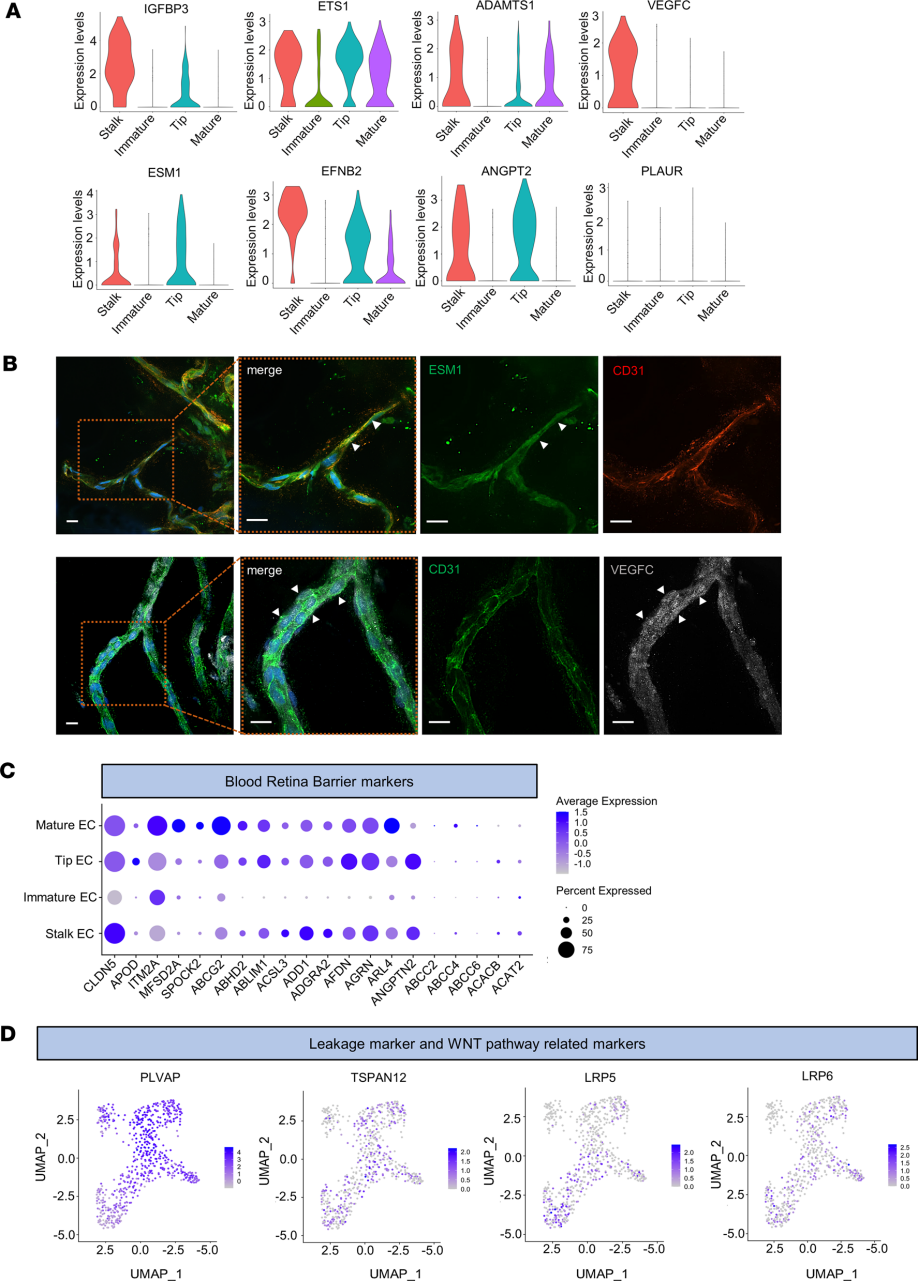
Endothelial cell gene expression profile analysis. (**A**) Violin plot for *IGFBP3*, *ETS1*, *ADAMS15*, *VEGFC*, *ESM1*, *EFNB2*, *ANGPT2*, and *PLAUR*, which are among the most expressed genes, in each endothelial cell cluster. (**B**) Representative images of immunofluorescence experiments on PDR membranes for ESM1 (upper) and VEGFC (bottom). CD31 was used to stain the endothelial cells, and DAPI was used to counterstain the nuclei. Scale bar: 10 μm. Representative images from 3 independent experiments are shown. Arrowheads indicate the CD31^+^ESM1^+^ cells in the upper panel in **B**. They indicate VEGFC localization inside of the blood vessels in the bottom panel in **B**. Negative control images are shown in Supplemental Figure 2. (**C**) Dot plot showing the panel of genes expressed in endothelial cells forming the blood retina barrier (BRB). Cell type classification and the percentage and average expression of the specific cell markers are shown. (**D**) Feature plots showing BRB leakage/maintenance genes.

We further investigated the gene expression profile for the endothelial subclusters. Stalk cells showed the expression of expected enriched genes such as *IGFBP3*, *ETS1*, and *ADAMTS1*. In addition, we identified a subset of genes not physiologically expressed in stalk cells, such as *VEGFC*, normally involved in the formation of lymphatic vessels (14, 15), as shown in the violin plots in Figure 3A. Similarly, *ESM1* and the system *EFNB2/EPH4* are usually mainly expressed by tip cells, where they enhance VEGFA signaling pathway to promote the formation of new blood vessels. *ANGPTN2* was found expressed in stalk cells as well, even though it is physiologically involved in modulating the migration and chemotactic activity of tip endothelial cells, by activating Rac1 (16).

We next analyzed the gene expression profile of tip cells and detected expression of genes such as *IGFBP3*, usually highly expressed in stalk cells, and *ETS1*, which is normally absent in tip cells. In addition, tip cells did not express *PLAUR*, a gene that is physiologically tip enriched (Figure 3A).

Immunofluorescence on PDR membranes was performed to confirm the active angiogenetic process of these endothelial cells. As shown in Figure 3B, labeled with the endothelial canonical marker CD31, most of the endothelial cells coexpressed ESM1 in areas of active sprouting. The higher magnification shows colocalization of CD31 with ESM1 within these new sprouts in more detail. Next, we immunostained the membranes for VEGFC to explore the unusual expression of this growth factor as shown in scRNA-Seq. VEGFC was detected as a diffuse signal in endothelial cells, confirming its production (Figure 3B).

Next, we evaluated whether these endothelial cells showed conservation of functions normally ascribed to the retinal blood vessels from which they originated. We examined a panel of blood-retina barrier (BRB) genes normally expressed by mature endothelial cells. We found a subset of genes expressed at similar levels in tip, stalk, and mature cells, and we found an additional set of genes that were not expressed at all, as shown in the dot plot in Figure 3C. We further analyzed the expression of transcriptome specific to components of the BRB tight junctions and observed an alteration in the expression of these genes. In particular, we observed that *CLDN1*, *CGN*, and the angulin family member *ILDR2* were not expressed in any of the endothelial cell clusters, while *CLDN12*, *TJP2*, *OCLN*, the angulin family member *LSR*, and the β-catenin target *AXIN1* and *AXIN2* were expressed at low levels in all the endothelial cell clusters. In contrast, *TJP1* was highly expressed in all the endothelial cell clusters (Supplemental Figure 3). In addition, we found expression of the leakage marker *PLVAP* in all endothelial cells (Figure 3D), sustaining the hypothesis that those cells have lost their retina capillary phenotype. PLVAP specifically localizes to diaphragms of fenestrated endothelial cells such as the choriocapillaris (17, 18); it is typically expressed in vascular beds performing high filtration, secretion, or transendothelial transport. It is absent in endothelial cells where barrier properties are critical, such as the BRB. Interestingly, PLVAP is expressed in the BRB under pathological conditions, leading to barrier disruption (19). On the other hand, we detected upregulation of WNT pathway–related genes — such as *TSPAN12*, *LRP5*, and *LRP6* — involved in the maintenance of BRB homeostasis (Figure 3D). Signaling through the frizzled class receptor 4/LDL receptor–related protein 5–6/tetraspanin 12 (FZD4/LRP5–6/TSPAN12) receptor complex is, in fact, required for developmental vascularization and BRB formation (20).

### Immune cells in the fibrovascular membranes express proangiogenic genes.

**Figure 4:**
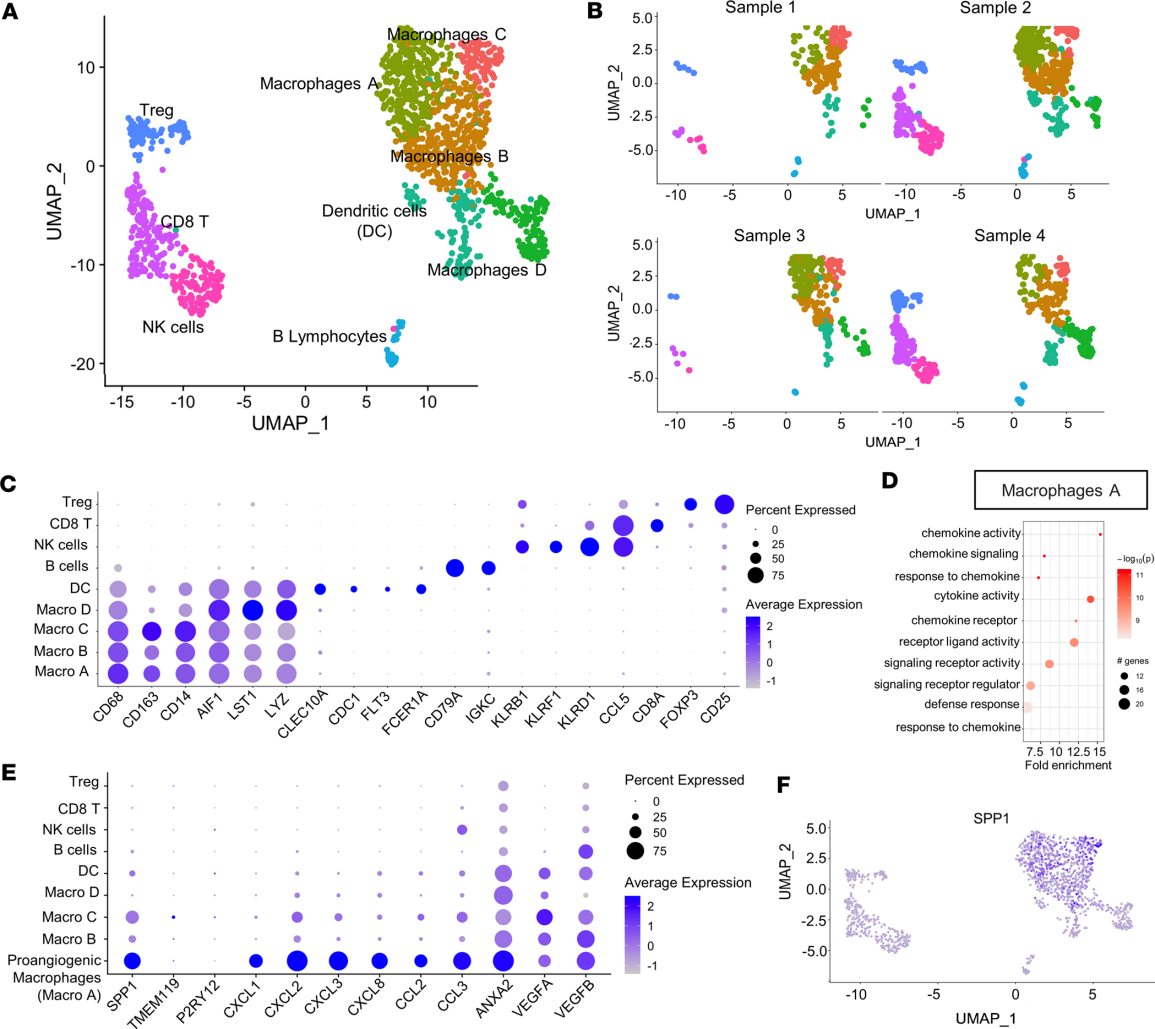
Immune cell clustering, gene expression profile, and macrophage-expressing proangiogenetic molecule identification. (**A** and **B**) Representative UMAP plot of the 9 different cluster of immune cells is shown in **A**, while UMAP plot for each sample is shown in **B**. (**C**) Dot plot for the most common markers for macrophages, DCs, NK cells, and B and T lymphocytes. Cell type classification and the percentage and average expression of the specific cell markers are shown. (**D**) Pathway enrichment analysis for the upregulated genes of Macrophage A cluster. Only the most significant differentially expressed genes (log_2_FC > 1 and *P*_adj_ < 0.01) were chosen for pathway enrichment analysis. The graph shows the number of genes modulated in each single pathway, the fold enrichment, and the statistical significance. (**E**) Dot plot of the proangiogenic cytokines, with the identification of proangiogenic *SPP1^+^* macrophages. Cell type classification the percentage and average expression of the specific cell markers are shown. (**F**) Feature plot of *SPP1*^+^ cell distribution is shown in **F**.

The importance of inflammation in the pathogenesis of DR is clear, and DR can be considered a chronic inflammatory disease (21, 22). In particular, macrophage-like cells are substantially increased on the retinal surface in human eyes with advanced DR (23–25). We focused on the immune cell clusters and identified 9 different clusters (Figure 4, A and B). To identify the cell type associated with each cluster, we relied on differentially expressed genes. *CD68*, *CD163*, *CD14*, and *AIF1* as well as *LST1* and *LYZ* allowed us to identify 4 macrophage clusters, and *CLEC10A*, *CD1C*, *FLT3*, and *FCER1A* were used to identify a DC cluster. The remaining 4 clusters were classified as lymphocytes. *CD79A* and *IGKC* were B cell markers; *KLRB1*, *KLRF1*, and *KLRD1* identified NK cells*; CCL5* and *CD8A* identified CD8 T cells; and *FOXP3* and *CD25* identified Tregs (Figure 4C).

It is interesting to note that CD8 T cells and NK cells were not evenly distributed among the 4 samples and were relatively underrepresented in samples 1 and 3, suggesting that these cells may not be critical to fibrovascular membranes.

Pathway enrichment analysis for the upregulated genes performed on the most expressed genes (log_2_FC > 1, *P*_adj_ < 0.01) allowed us to identify a particular cluster of macrophages, Macro A, where chemokine mediated signaling pathways and cytokine activity were among the most modulated pathways (Figure 4D). Deeper analysis of the genes in this biological process showed the expression of chemokines known to have proangiogenetic properties, such as *CXCL1*, *CXCL2*, *CXCL3*, *CXCL8*, *CCL2*, and *CCL3* (Figure 4E). This cluster (Macrophage A), which we labeled as proangiogenic macrophages, expressed the activated macrophage marker *SPP1* and did not express microglia markers such as *TMEM119* and *P2RY12* (Figure 4, E and F). When compared with cluster A, the expression of *SPP1* was relatively lower in clusters B and C, with dissimilar expression levels between these 2 clusters. Other immune cell clusters did not express the same panel of chemokines, but they participated in the angiogenetic process by producing other growth factors, such as *ANXA2*, *VEGFA*, and *VEGFB*. Ligand-receptor analysis confirmed the interaction between immune and endothelial cells. In particular, we found that macrophages communicate with the endothelial cells by transcribing molecules related to the Notch pathway, well-known to be essential for angiogenesis, in addition to the CXCL/CCL family chemokines and VEGF molecules (Supplemental Figure 4). It is worth noting that the inflammatory cells, whether macrophages or T cells, sustain a crosstalk with stromal cells as well, further supporting their potential interactions that promote the fibrovascular membranes (Supplemental Figure 4).

**Figure 5:**
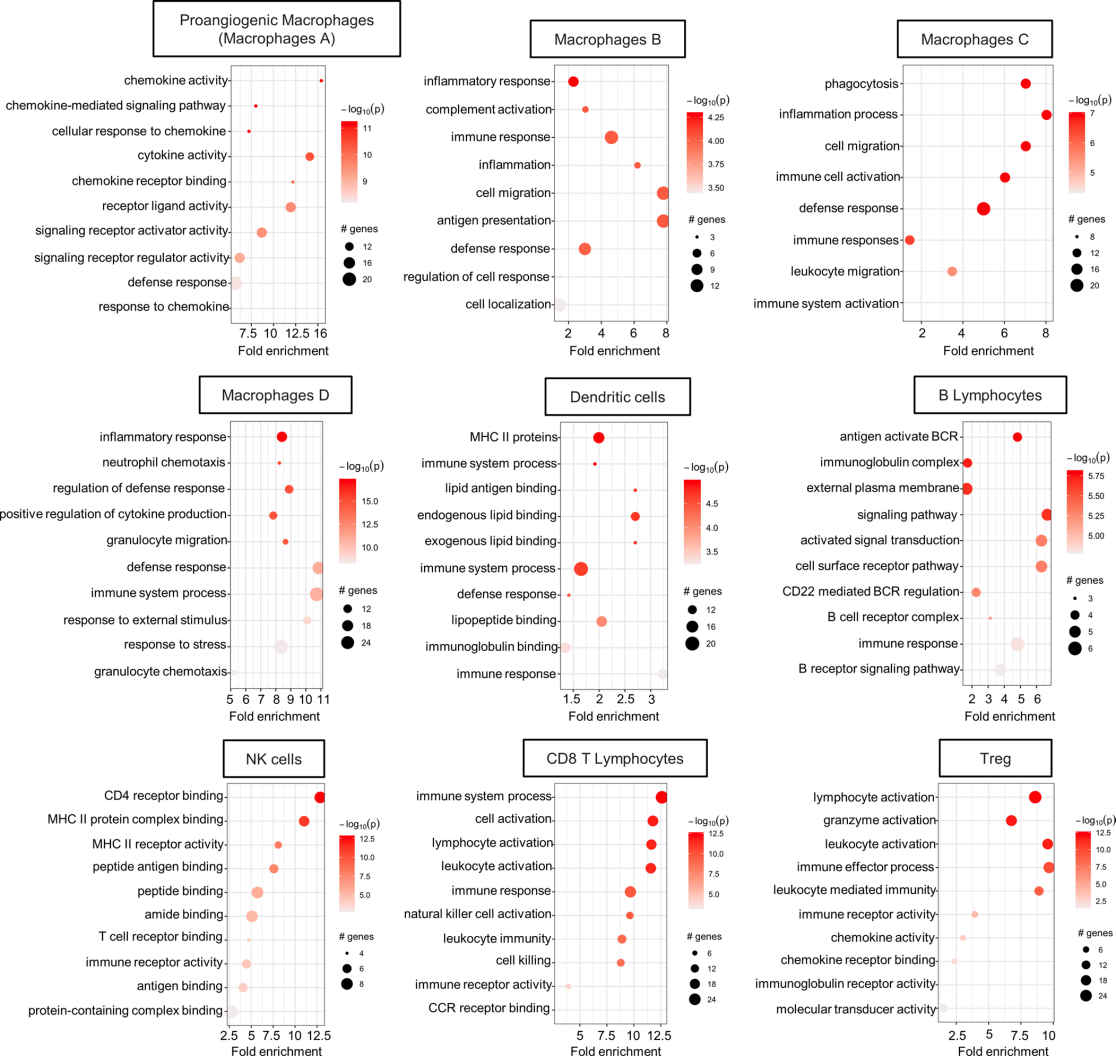
Immune cell pathway enrichment analysis for the upregulated genes. Dot plot of the pathway enrichment analysis for the upregulated genes of each cluster using gProfiler and PathFindR package is shown. Only the most significant differentially expressed genes (log_2_FC > 1 and *P*_adj_ < 0.01) were chosen for pathway enrichment analysis. The graph shows the number of genes modulated in each single pathway, the fold enrichment, and the statistical significance.

Pathway enrichment analysis of the clusters for the upregulated genes showed processes primarily related to specific functions of immune cells (Figure 5). Inflammatory response, phagocytosis, migration processes, granulocytes, and neutrophil chemotaxis were mainly enriched in the macrophage clusters. Enriched pathways related to MHCII activation and antigen-presenting processes were enriched in DC clusters. Pathway analysis in lymphocyte clusters revealed the activation of processes related to B cell receptor signal transduction in B lymphocytes; CD4 receptor activity in NK cells; and granzymes, cell activation, cell killing were, as expected, enriched in CD8 T cells, and lymphocyte activation–related pathways were enriched in Tregs. Pathway enrichment analysis for the downregulated genes (Supplemental Figure 5) confirmed the identity of each cluster but did not reveal particularly unexpected pathways.

These results corroborate the important role of immune cells in fibrovascular membrane formation, showing the expression of a variety of macrophage-derived inflammatory cytokines and growth factors with proangiogenic functions.

### Identifying the pericyte cluster within the stromal cells and defining their role in fibrogenesis.

**Figure 6:**
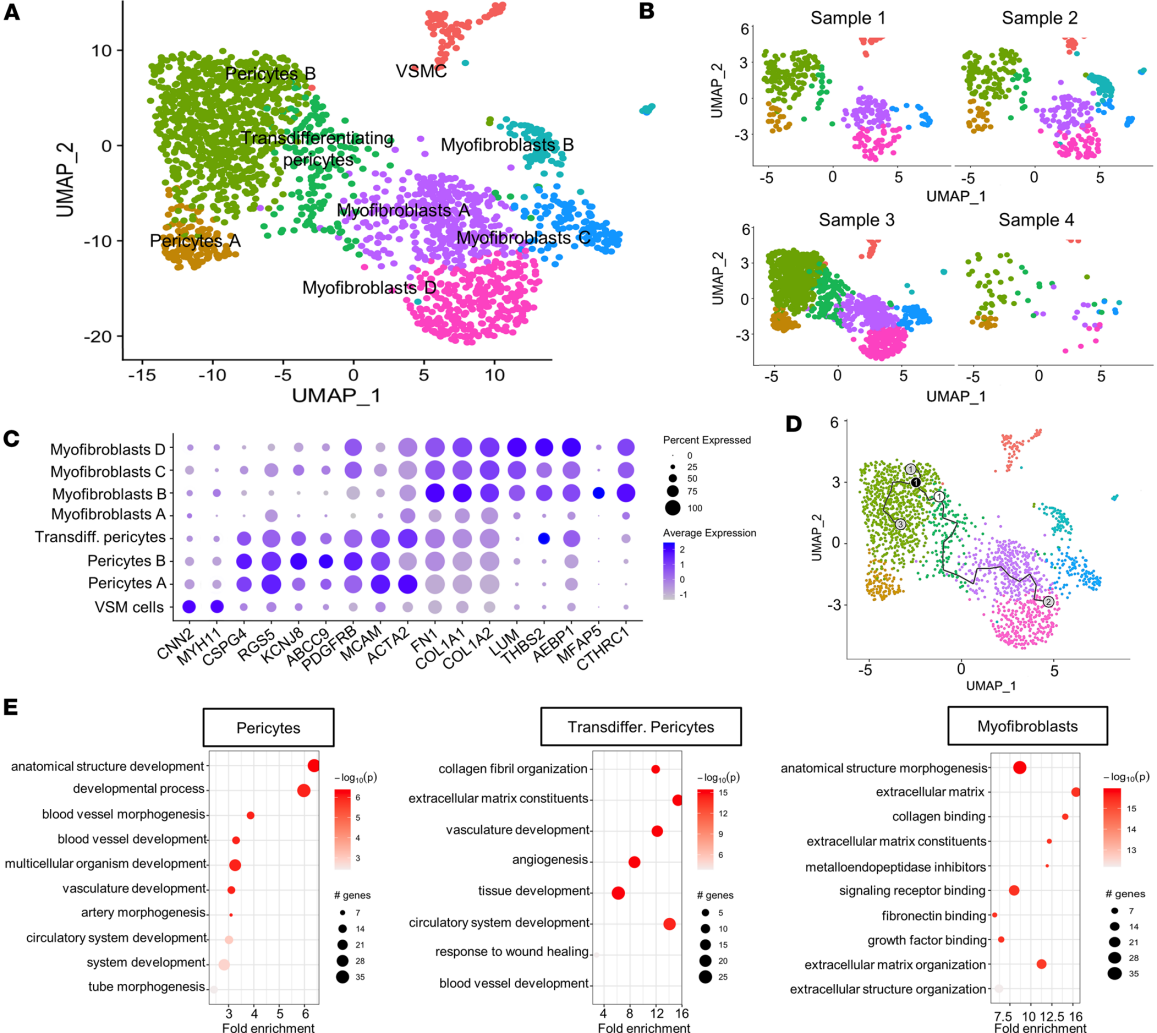
Stromal cell clustering, gene expression profile, and cell trajectory inference analysis. (**A** and **B**) Representative UMAP plot of the 8 different cluster of stromal cells is shown in **A**, while UMAP plot for each sample is shown in **B**. (**C**) Dot plot for the most common markers for pure pericytes and myofibroblasts. Cell type classification and the percentage and average expression of the specific cell markers. (**D**) Trajectory inference analysis of the stromal cell clusters. The crucial node of the pseudotime analysis was identified in the transdifferentiating pericyte cluster, with 2 branches starting from this point — 1 of them toward myofibroblast lineage. (**E**) Dot plot of the pathway enrichment analysis for the upregulated genes of representative cluster of pericytes, transdifferentiating pericytes, and myofibroblasts, using gProfiler and PathFindR package. Only the most significant differentially expressed genes (log_2_FC > 1 and *P*_adj_ < 0.01) were chosen for pathway enrichment analysis. The graph shows the number of genes modulated in each single pathway, the fold enrichment, and the statistical significance.

We next wondered if the newly formed blood vessels were covered by supporting cells that normally contribute to their function and homeostasis. We reclustered the stromal cells and identified 8 clusters (Figure 6, A and B). Based on differential gene expression, we found 1 cluster of vascular smooth muscle cells, expressing *CNN2* and *MYH11*, but the rest of the cells were divided in 2 main groups. In the first group, we identified 2 clusters of pericytes, expressing a panel of canonical genes such as *CSPG4*, *RGS5*, *KCNJ8*, *ABCC9*, *PDGFRB*, and *MCAM* (Figure 6C), while the second group expressed myofibroblastic genes such as *ACTA2*, *FN1*, *COL1A1*, *COL1A2*, *LUM*, *THBS2*, *AEBP1*, *MFAP5*, and *CTHRC1*. Myofibroblasts are the cells responsible for the production of collagen and other extracellular matrix components, which are also the building blocks of scar tissue and fibrosis (Figure 6C). Interestingly, we identified an intermediate cluster between pericytes and myofibroblasts, which expressed genes overlapping with both cell types, suggesting that cells within this cluster were transitioning from their pericyte origin toward a myofibroblastic identity (Figure 6C).

Next, we performed cell-inference trajectory analysis (or pseudotemporal ordering) to evaluate the dynamic process of transformation experienced by the cells and to identify the intermediate cell stages during this process. The analysis, reported in Figure 6D, showed that the crucial node 1 was in the transdifferentiating cluster, which we identified as the source of myofibroblasts. From this crucial node, 2 different branches emerged, describing 2 different possible dynamics. One of these branches connected with the pericyte clusters, which maintain canonical gene expression; the other branch connected to myofibroblastic clusters, with a shift in the gene expression profile, lending further support to the idea that a pericyte subset transformed toward myofibroblastic lineage (Figure 6D).

Pathway enrichment analysis for the upregulated genes reinforced this hypothesis by showing that pericytes were enriched in pathways related to physiological functions, such as blood vessel morphogenesis and development. In contrast, the transdifferentiating cluster showed pathways related to extracellular matrix components in addition to the canonical pericyte function pathways, suggesting activation of fibrogenic signaling pathways (Figure 6E). Enrichment pathway analysis in myofibroblasts showed activation of processes related to extracellular matrix components, collagen binding, extracellular matrix organization, and fibronectin binding. The pathway enrichment analysis for the downregulated genes further corroborated these results. As expected collagen binding and extracellular matrix genes were downregulated in the pericyte clusters, and genes related to angiogenesis and blood vessel development were downregulated in the myofibroblasts. The transdifferentiating pericyte cluster showed downregulation of genes related to vasculature development and the *PDGFR* signaling, a canonical pericyte pathway, confirming partial loss of pericyte identity (Supplemental Figure 6).

Notably, ligand receptor analysis (Supplemental Figure 4) showed an interesting cross-talk between myofibroblasts and endothelial cells through VEGFC signaling, in addition to the Notch pathway. This could be an interesting, previously unrecognized mechanism whereby VEGFC-expressing, pathological endothelial cells might promote the myofibroblast phenotype. In addition, myofibroblasts expressed molecules of the CCL/CXCL pathway and other members of the VEGF pathways as well as additional proangiogenic factors.

### AEBP1 as a key marker of retinal pericyte transition toward myofibroblastic phenotype.

**Figure 7:**
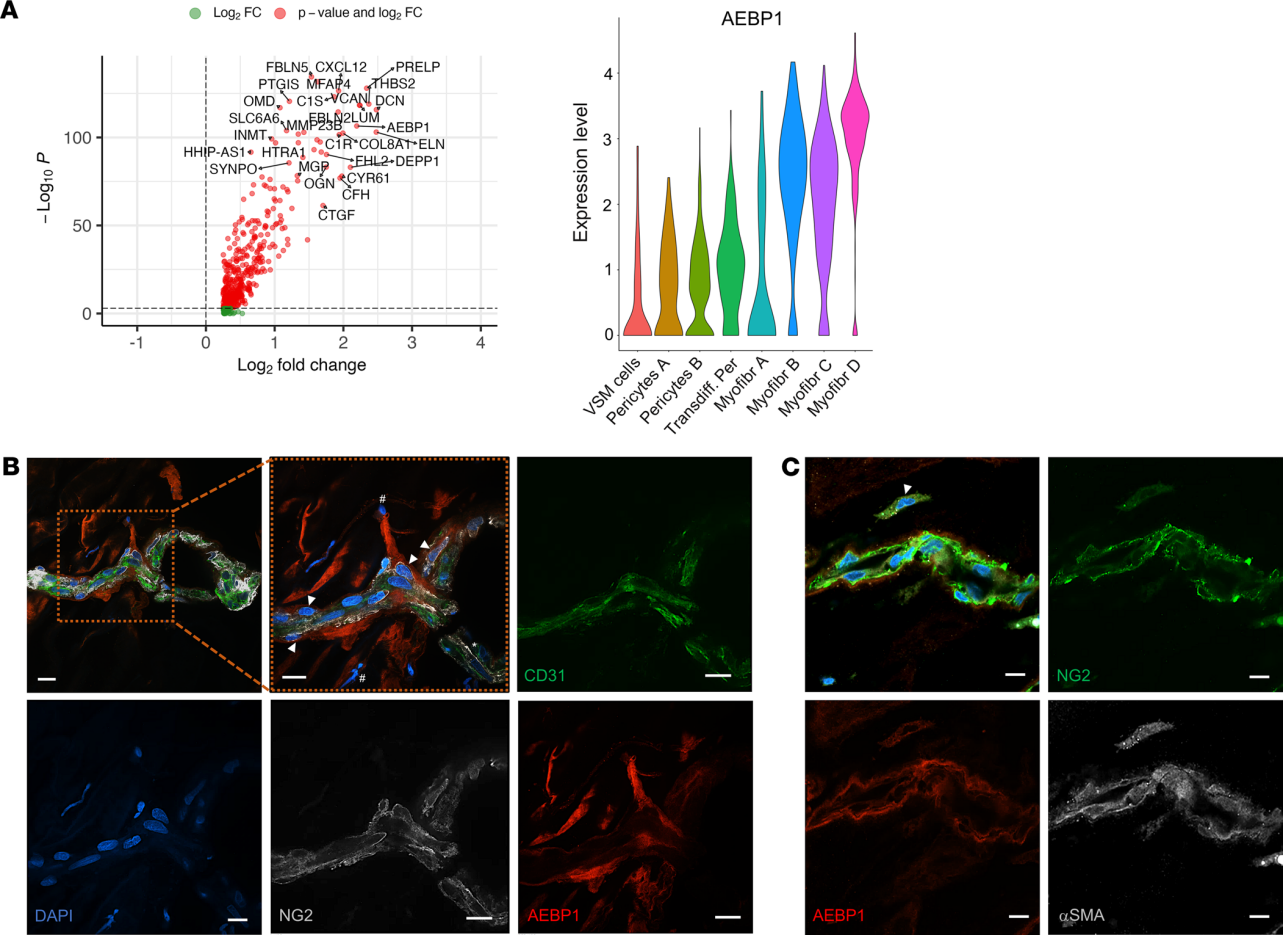
*AEBP1* gene identification in the stromal cluster. (**A**) Volcano plot showing the top differentially expressed genes in the myofibroblast clusters and violin plot for *AEBP1* expression in all the stromal cell clusters. (**B**) Immunofluorescence on PDR membranes for AEBP1 and the pericyte marker NG2. CD31 was used to visualize the endothelial cells, and DAPI was used to counterstain the nuclei. (**C**) Immnostaining for AEBP1, NG2, and αSMA. Scale bars: 10 μm. Arrowheads indicate NG2^+^AEBP1^+^ cells around the blood vessels; the pound symbols indicate AEBP1^+^ cells outside of the blood vessel area and the asterisk indicates NG2^+^ cells. Representative images from 3 independent experiments are shown. Negative control images are shown in Supplemental Figure 2.

To better clarify the molecular mechanism driving the pericyte transition toward myofibroblastic phenotype and to find molecules that might be critical for this process, we analyzed the differential gene expression in the stromal clusters. Among the top 10 most differentially expressed genes, we found *THBS2*, *LUM*, *CTGF*, *COL8A1*, *VCAN*, and *FBLN5* significantly higher in the myofibroblasts when compared with pericytes. Most of these are well-known extracellular matrix proteins that are downstream of fibrogenic signaling pathways. *Adipocyte enhancer-binding protein 1* (*AEBP1*), a profibrotic molecule and transcription factor, previously identified in a variety of tissues but not in preretinal fibrosis in human PDR, was also significantly upregulated in the myofibroblastic clusters when compared with the pericyte clusters, as shown in the volcano plot and in the violin plot in Figure 7A. We therefore hypothesized that AEBP1 might be one of the key molecules contributing to the pericyte change toward myofibroblastic phenotype in this context.

Immunofluorescence of PDR membranes showed that AEBP1 was expressed in most of the NG2^+^ pericyte cells surrounding endothelial cells stained with CD31, reflecting the molecular change of these pericytes (Figure 7B). It is worth mentioning that some of those transitioning pericytes had slightly different morphology, appearing as elongated mesenchymal cells, highlighted with arrowheads. As expected, some of the NG2^+^ cells did not stain with AEBP1, suggesting that those pericytes retained their physiological gene expression and function (Figure 7B). Moreover, AEBP1 expression was also observed in other cells, not associated with the blood vessels and not expressing NG2 (Figure 7B). We cannot confirm the nature of these cells, which could be fully transdifferentiated toward myofibroblast lineage, or other cell types, unrelated to pericytes. We also stained the membranes with the myofibroblast marker αSMA and found that AEBP1^+^NG2^+^ pericytes were also αSMA^+^ and were localized outside of the blood vessel area; they were presumably detached pericytes undergoing transformation (Figure 7C).

**Figure 8:**
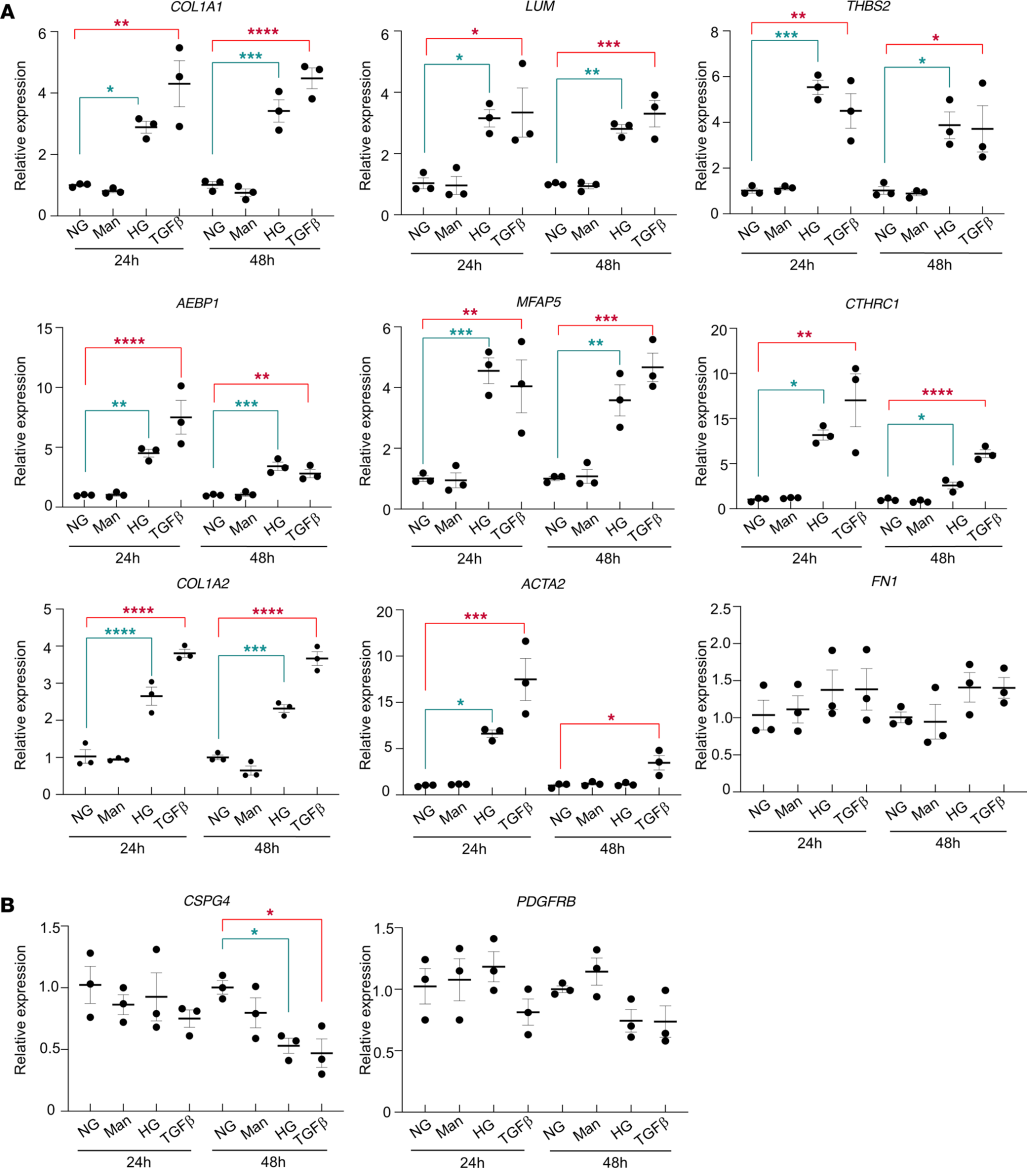
Molecular changes in Human Retinal Pericytes (HRP) cultured in high-glucose medium. (**A** and **B**) mRNA expression for profibrotic genes *COL1A1*, *COL1A2*, *LUM*, *THBS2*, *AEBP1*, *CTHRC1*, *MFAP5*, *ACTA2,* and *FN1* (**A**) and pericyte markers *CSPG4* and *PDGFRB* (**B**) upon 24-hour and 48-hour high glucose (30 mM) medium culture. TGF-β treatment (10 ng/mL) was used as a positive control for fibrosis induction and mannitol (24nM) was used as osmotic control. Summary of 3 independent experiments is shown. One-way ANOVA followed by Tukey’s multiple comparison test were used. *P < 0.05, **P < 0.01, ***P < 0.001, ****P < 0.0001. NG, normal glucose (*n* = 6); Man, mannitol (*n* = 6); HG, high glucose (*n* = 6); TGF-β (*n* = 6).

To better characterize the molecular effects of the diabetic microenvironment on pericytes and corroborate the human fibrovascular PDR membranes findings, we cultured human retinal pericytes (HRP) in high-glucose medium for 24 and 48 hours and compared these with cells in osmotic control and normal media. Quantitative PCR (qPCR) analysis in HRP showed significant upregulation of profibrotic markers, such as *COL1A1*, *COL1A2*, *LUM*, *THBS2*, *MFAP5*, *CTHRC1*, and *ACTA2*, as early as 24 hours of high glucose. This upregulation was maintained for most of the genes at 48 hours of culture. We next used TGF-β stimulation as a positive control for its induction of the fibrotic phenotype (Figure 8A). *AEBP1* gene expression was significantly upregulated as well, confirming the PDR membrane scRNA-Seq and immunofluorescence results (Figure 8A). We then analyzed the canonical pericyte genes at the same time points in culture. After 48 hours in high glucose, *CSPG4*, encoding for NG2, showed significant decline, suggesting the onset of pericyte cell identity loss (Figure 8B).

**Figure 9:**
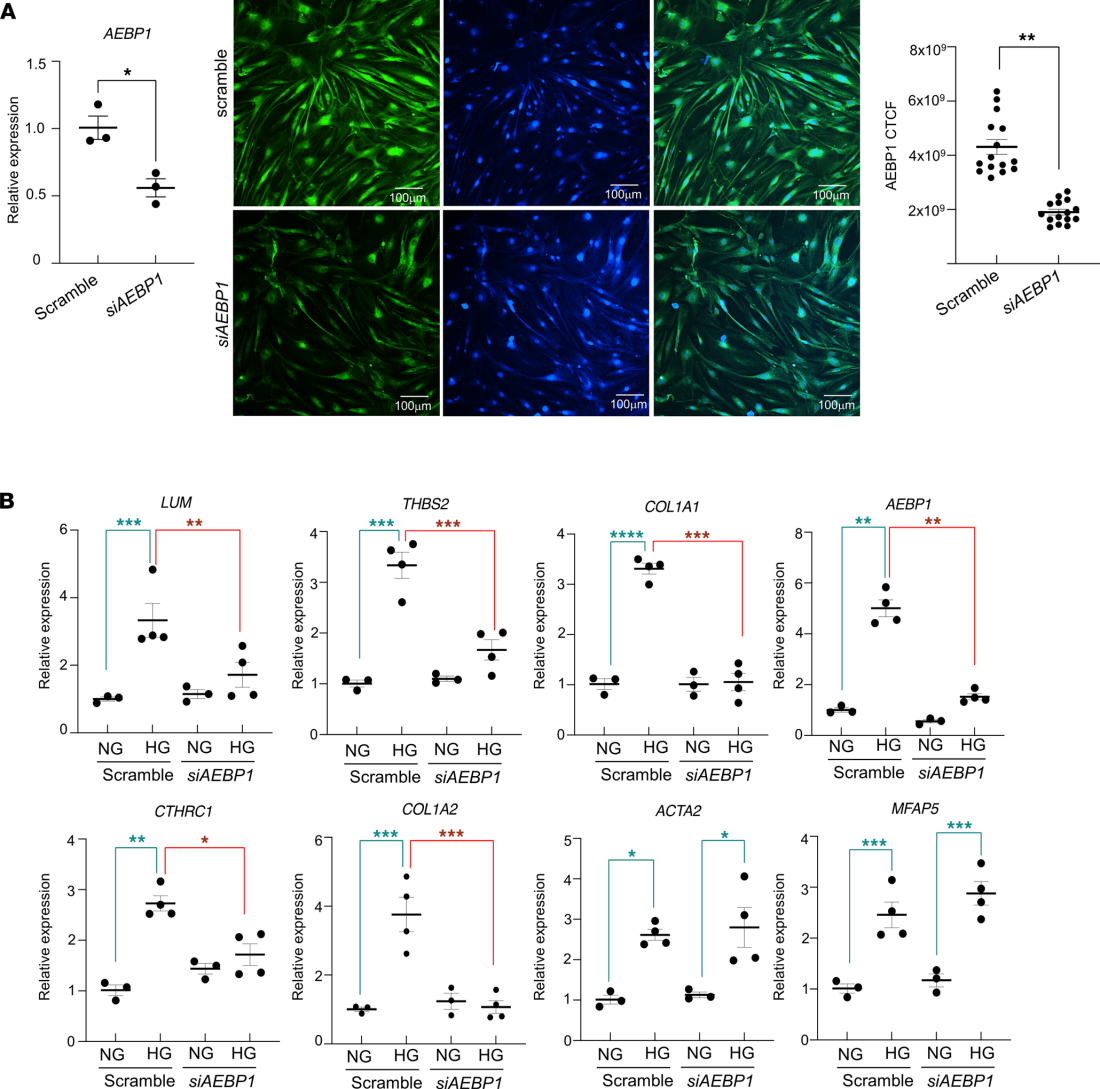
Molecular changes in human retinal pericytes (HRP) cultured in high-glucose medium after *siAEBP1* transfection. (**A**) mRNA and protein expression for AEBP1 after siRNA transfection. Scale bar: 100 μm. Representative images from 3 independent experiments are shown. (**B**) mRNA expression for profibrotic genes *COL1A1*, *COL1A2*, *LUM*, *THBS2*, *CTHRC1*, *MFAP5*, *ACTA2*, and *AEBP1* upon 24-hour culture in high glucose (30 mM) medium, with downregulation of endogenous *AEBP1* levels by siRNA assay. Summary of 3 independent experiments is shown. Two-way ANOVA test and Mann-Whitney *U* test were used. *P < 0.05, **P < 0.01, ***P < 0.001, ****P < 0.0001. NG, normal glucose (*n* = 6); HG, high glucose (*n* = 8).

Next, to determine whether blocking *AEBP1* expression could protect pericytes against the fibrogenic effect of high glucose, we downregulated *AEBP1* expression by siRNA in HRP cultured in normal or high glucose. After confirming siRNA-mediated downregulation of AEBP1 mRNA and protein expression (Figure 9A), we observed significantly decreased profibrotic gene expression in HRP cells exposed to high glucose when transfected with *AEBP1* siRNA compared with cells transfected with nontargeting siRNA (scramble). Notably, we observed a substantial attenuation of *COL1A1*, *LUM*, *THBS2*, *AEBP1*, *COL1A2*, and *CTHRC1*, but not of *MFAP5* and *ACTA2* expression (Figure 9B). These results confirmed the involvement of *AEBP1* in driving and modulating the pericyte transition toward myofibroblastic phenotype.

## Discussion

In this study, we highlight important concepts in the pathogenesis of fibrovascular membranes in advanced PDR. We show that pathological endothelial cells undergo active angiogenesis with a pathological gene expression profile; macrophages express proangiogenic genes, potentially contributing to the angiogenic process; and pericyte trajectory and molecular analysis suggest partial loss of their pericyte identity and a transformation process toward myofibroblastic phenotype. Using scRNA-Seq allowed us to identify the main cell populations in surgically removed fibrovascular membranes and to understand the molecular pathways that regulate these processes.

Pathological neovascularization is the hallmark of PDR (26, 27). It is a multistep process, where endothelial cells play a central role with contributions from a variety of other cell types. Various events are necessary for angiogenesis to occur, including the interaction between cell surface receptors, soluble factors, and extracellular matrix components (16). A change in the microenvironment, usually hypoxia or oxidative stress in diabetes, triggers this cascade of events, promoting the release of the master proangiogenetic molecule, VEGFA. This induces a change in the permeability of blood vessels and a switch of the endothelial cell profile from “quiescent” toward a “proangiogenic” phenotype, and these cells proliferate and migrate into the stroma toward a chemotactic gradient provided by the angiogenic stimulus (16). The specialized endothelial cells responsible for initiating this process are called tip cells. They sense the environment and emit filopodia to initiate the spouting of a new blood vessel, followed by other specialized cells, the stalk cells, that drive the spouting and the elongation of the vessel (16). During the dynamic process of pathologic angiogenesis, endothelial cells show a wide range of transcriptomics plasticity across tissues and diseases, allowing these cells to adapt to the microenvironment of the diverse pathologies. In this context, Rohlenova et al. compared the transcriptome of pathological endothelial cells in rodent choroidal neovascularization (CNV) and murine lung tumor models (13). They identified an interesting cluster of proliferating cells, distinct from immature, tip, and neophalanx endothelial cells. The proliferating cells across disease models showed high expression of cell cycle regulation and cell-division molecules, including single-carbon pathways. Importantly, these authors emphasized similarities as well distinct metabolic pathway differences between endothelial cells in tumor and CNV models, suggesting important disease- and tissue-related adaptations during pathologic angiogenesis. Our results show similar endothelial cell phenotypes in the fibrovascular PDR membranes, including tip, stalk, mature, and immature cells (10, 12), but we did not detect a unique cluster of proliferating cells. Instead, we found that gene expression related to proliferation was similar among all endothelial subclusters, with relatively lower expression in the immature subcluster. Studying the endothelial metabolic pathway gene expression, we found that glycolysis-related genes showed generally higher expression compared with the tricarboxylic acid cycle genes in all endothelial clusters, with glycolysis being most highly expressed in the immature cell compartment. In our PDR membranes, the immature endothelial cells had the closest metabolic profile to the proliferating endothelial cell cluster from rodent CNV, including the highest glycolytic, nucleotide biosynthesis, and oxidative phosphorylation activity. Uniquely, these cells had the lowest expression of proliferation markers, differentiating them from the proliferating cluster in rodent CNV endothelial cells that had the highest proliferation activity. We considered a variety of potential explanations for these metabolic differences in angiogenic endothelial cells, including tissue microenvironment adaptation (choroid versus retinal angiogenesis), strain differences (mouse versus human), or true pathologic differences related to the diabetic microenvironment, the chronicity, and/or fibrotic nature of these PDR membranes.

We found that the endothelial cells in our samples were actively involved in the angiogenic process, as shown by the enrichment pathway analysis and confirmed by immunofluorescence for ESM1, a tip cell marker that colocalized with CD31 in the sprouting sites on these PDR membranes. Our analyses also showed that these endothelial cells not only expressed their canonical genes but also displayed transcriptomics profile irregularities, highlighting their pathological nature. The presence of VEGFC in the stalk cell compartment was unexpected, since it is normally expressed and involved in lymphatic vessel development (14, 15, 28).

The potential cooperation of all the VEGF family members during retinal neovascularization allows them to establish a regulatory network. For example, VEGFA promotes the expression of VEGFC and VEGF receptor 3 (VEGFR3) in human retinal pigment epithelial cells (29). VEGFC and VEGFD also bind and activate VEGFR2 independently of VEGFA, and they are the only ligands for VEGFR3 (30, 31).VEGFs are therefore potent modulators of neovascularization, and the anti-VEGF molecules, which mostly target VEGFA (except aflibercept, which also targets VEGFB and placental growth factor), have emerged as important therapies that address leakage and angiogenesis in a variety of retinopathies, including DR (6, 32, 33). These medications fail in a high percentage of eyes, which prompted an ongoing active search for escape mechanisms and alternative druggable pathways (26, 33). Therefore, it is plausible that VEGFC induction potentiates the VEGFA signaling pathway and the pathological neovascularization in DR and that it may represent a potential mechanism for anti-VEGFA nonresponsiveness. VEGFC has been described as involved in promoting angiogenesis in vivo, is sufficiently potent to stimulate neovascularization from limbal vessels in the mouse cornea, and is able to induce branch sprouts from established blood vessels in the developing embryo. In addition, VEGFC can induce actin reorganization and change in the cell morphology in both VEGFR2- and VEGFR3-overexpressing endothelial cells (34). Moreover it is overexpressed, together with its receptor and other molecules, in neovascularization in PDR. High-throughput sequencing of 23 eye donors with DR and surgical samples from PDR eyes showed that the transcriptomics profile of surgical neovascular membranes from PDR samples was distinct from donor diabetic retina and that VEGFC, VEGFR2, VEGFR3 were specifically upregulated in neovascular membranes (35). The ligand-receptor analysis in our study further showed that the VEGFC pathway was involved in endothelial cell–myofibroblast crosstalk, revealing another potential mechanism whereby this growth factor might contribute to fibrovascular membrane formation. Targeting VEGFC could therefore improve anti-VEGF treatment responsiveness and curb myofibroblastic proliferation, a possibility that is currently under investigation in ongoing clinical trials (36).

We next explored the functional genes of endothelial cells and found abnormally low expression levels of several critical BRB genes — including *ANGPT2* and most of the tight junctions such as *CLDN1*, *CLDN12*, *OCLN*, *CNG*, *TPJ2*, *ILDR2*, and *AXIN1-2* — and upregulation of leakage markers, such as *PLVAP* in all endothelial cell types, consistent with the pathological nature of these endothelial cell in newly formed blood vessels. PLVAP has been shown to be upregulated in the BRB in pathological conditions, whereas physiologically, it is only expressed in fenestrated endothelial beds and not in those with a critical barrier functions (17–19). Interestingly, a recent study showed that loss of endothelial angiopoietin 2 was responsible for abnormal brain vascular morphology, increased vascular leakage, abnormalities of endothelial cell shape, and alteration of the distribution of claudin 5, an important tight junction of the blood-brain barrier (37). This study would suggest that the focal absence of molecules normally distributed in the barrier may be sufficient to impair the distribution of other molecules and disrupt the overall barrier function. We suspect that similar dysfunction may translate to the retinal BRB as well.

Inflammation is considered a central pathology in DR, manifesting as increased serum as well as vitreous and aqueous humor inflammatory cytokines and chemokines (21, 22). Inflammation has been proposed as one the first molecular events in DR pathogenesis leading to accumulation of inflammatory mediators and potentially contributing to neuronal cell death in the retina (22, 38). Moreover, inflammation and neovascularization have an established crosstalk, with inflammation promoting neovascularization and vice versa (39). The use of corticosteroids as an alternative to anti-VEGF therapy targets inflammation but may also interrupt the positive feedback with angiogenesis in endothelial cells (40). In our data set, ligand-receptor analysis showed crosstalk between endothelial and immune cells, in particular with macrophages, an interaction that is driven by the Notch, CXCL/CCL, and VEGF pathways. Interestingly, macrophages in our data sets showed crosstalk with stromal cells as well, and this could be playing an important role in these fibrovascular processes. Moreover, our data show multiple types of inflammatory cells, including macrophages, DCs, B cells, NK cells, and T cells, suggesting these fibrovascular membranes sustain an active inflammatory process. Among the immune cells, we identified a cluster of macrophages, *SPP1*^+^ cells, expressing a host of proangiogenetic genes, therefore potentially contributing to and exacerbating the angiogenetic environment (41). It is worth noting that none of the inflammatory cells expressed microglia markers, such as *TMEM119* and *P2RY12*, in contrast to recent work that claimed microglial population as a main cell type involved in the fibrovascular membrane formation in PDR, with a subpopulation of microglia presenting fibrogenic properties (42). When we accessed the online deposited raw data from this work, we were unable to replicate their conclusion in their own data sets. The majority of immune cells in their study were macrophages, with very low expression levels of microglial markers such as *TMEM119* and *P2RY12*. Importantly, these immune cells did not show any transcriptomics signature relevant to fibrosis. Reassuringly, we were able to validate the fibrogenic gene profile in the stromal cell cluster in their data set, including the intermediate transdifferentiating pericyte cluster (specifically *CSPG4^+^MCAM^+^* pericytes), lending further support to our current study (Supplemental Figure 7).

The stromal compartment revealed interesting findings regarding fibrosis, a late-stage complication of DR that eventually leads to retinal detachment and loss of vision. Among the highly expressed genes in the stromal clusters, *AEBP1* was significantly upregulated in the myofibroblast compared with the pericyte cluster. *AEBP1* encodes a member of carboxypeptidase A protein family. The encoded protein was initially described as a transcription factor that negatively regulates the adipose cell fate, by tipping the balance away from adipogenesis toward fibrogenesis, promoting these cells to switch phenotype toward myofibroblasts (43). This distinct mechanistic role makes it an interesting molecule to investigate and potentially target therapeutically. Subsequent studies suggested that AEBP1 exerts its function via a variety of proposed mechanisms, as a WNT pathway ligand (44, 45), inducing the PPARγ pathway (46), and enhancing the TGF-β pathway (47). This protein appears to be extremely adaptable to the microenvironment where it exerts its functions. In addition, the crosstalk between AEBP1 and TGF-β, a potent fibrogenic promoter (48), is consistent with its proposed role in fibrogenesis. Previous studies have implicated AEBP1 as a fibrogenic factor in multiple organs, including the heart, lung, liver, adipose tissue, pancreas, and keloid formation (45, 49–52). Several studies showed that AEBP1 expression correlates with severity of fibrosis in nonalcoholic steatohepatitis (NASH), where hepatic expression of the protein exacerbates the disease both in humans and mice (45, 49, 53). It has also been shown to be expressed in lung fibrosis, and its absence protects against bleomycin-induced lung fibrosis (52). Lin et al. sequenced 6 keloid dermis and compared them with control dermis, showing that AEBP1 was among the candidate core regulators that promote the bone/cartilage-like characteristics in keloids. In addition, RNA-Seq of full-skin keloids and IHC experiments consolidated the role of this molecule (51). AEBP1 also enhances adipose tissue stromal progenitor differentiation into myofibroblasts and is upregulated in fibrotic white adipose tissue (50). We now expand the range of this molecule to preretinal fibrosis in human PDR.

*AEBP1* in our study showed increased gene expression in a subset of pericytes, which we labeled transdifferentiating pericytes, that expressed a combination of pericyte and myofibroblast genes. We hypothesize that this cluster is in the process of transition from one identity to the other. Pseudotime analysis of the stromal cells confirmed that the “transdifferentiating” cluster represented the crucial node. In this cluster, *AEBP1* expression was higher than in other pericyte clusters and showed a positive trend toward myofibroblasts. Our findings are in agreement with studies that have implicated pericytes as a source of myofibroblast progenitors in fibrosing organs (54, 55) and pericyte-myofibroblast transformation in a mouse model of scleroderma (56).

Immunostaining of fibrovascular PDR membranes supported the involvement of a subset of pericytes, coexpressing NG2 and AEBP1, in the transformation process. Those cells were also positive for the myofibroblast-enriched gene αSMA. Confirmatory in vitro experiments using HRP showed that a high-glucose environment induced pericyte expression of genes related to myofibroblastic phenotype. Moreover, blocking *AEBP1* suppressed these myofibroblast-like molecular changes.

Limitations of our study include the small sample size, which may reflect a general limitation of these pathological cells’ ability to withstand the technical process of single-cell isolation. We are encouraged that 4 PDR fibrovascular membranes yielded a reasonable number of cells (4,044 cells) with excellent viability, comparable with similar studies (42), which allowed subsequent sequencing and further analysis. Also, the absence of a healthy “control” sample is another critical point. Since we are studying a pathologic structure (diabetic fibrovascular membranes) that does not exist in the healthy eye, comparisons to human retinal or choroidal tissue would be the next best alternative for pericytes and endothelial cells. We explored existing human scRNA data sets (57, 58), but unfortunately, these data sets had very limited healthy retinal endothelial cells and even fewer pericytes, precluding our ability to perform robust comparisons.

In summary, our results support the hypothesis that AEBP1 is a crucial molecule promoting pericyte transition toward myofibroblastic phenotype and preretinal fibrosis in PDR. This molecule could be targeted to improve the visual outcome in patients with complications of advanced PDR, an important unmet need. Further studies will be necessary to investigate the molecular mechanism underlying AEBP1 function and to further address its therapeutic potential in vivo.

## Methods

### Collection and dissociation of fibrovascular membranes.

All human samples were collected through an approved IRB protocol from Northwestern University (detailed information about the patients are reported in the Supplemental Table 1). After surgery, PDR fibrovascular membranes were placed in balanced salt solution (BSS) and stored on ice for transportation. For dissociation, membranes were transferred to a 500 μL solution of 0.22 µm filter sterilized 1× Dulbecco’s PBS (DPBS) with calcium and magnesium (Invitrogen) containing 2 mg/mL Collagenase Type I (Worthington). Samples were incubated at 37°C for 30 minutes with gentle trituration every 10 minutes. Samples were then spun down at 400*g* for 8 minutes at 4°C, and cell pellets were resuspended in 500 μL 1× DPBS without calcium and magnesium (Invitrogen), 1% BSA, and 2 mM EDTA and stored on ice until further processing. Since membrane size varied from patient to patient, cell counts were not always robust enough to permit processing of all genetic profiling and/or immunofluorescence techniques listed below.

### scRNA-Seq.

All scRNA-Seq experiments were performed at the Functional Genomics Core at the University of Chicago (Chicago, Illinois, USA). Single-cell libraries were generated using a Chromium Single Cell 3′ v3 kit (10x Genomics). RNA quality and quantity were assessed using an Agilent Bio-analyzer. RNA-Seq libraries were prepared using Illumina mRNA TruSEQ kits following manufacturer instructions (Illumina). Library quality and quantity were checked using an Agilent Bioanalyzer, and the pool of libraries was sequenced using an Illumina HiSEQ4000 (paired-end 100 bp) using Illumina reagents and protocols.

Raw sequencing data, in base call format (.bcl) was demultiplexed using Cell Ranger (version 4.0.0) from 10x Genomics, converting the raw data into FASTQ format. Cell Ranger was also used for aligning the FASTQ files to the mm10 reference genome provided by 10x Genomics and for counting the number of reads from each cell that align to each gene. The matrix files and feature files summarizing the alignment results were analyzed in Seurat (Satija Lab, New York Genome Center [NYGC], New York, New York, USA) for further analysis. In Seurat, each individual sample was preprocessed, normalized, and scaled. Each sample underwent quality control measures to check for the number of genes, UMIs, and percent mitochondrial genes detected per cell, and appropriate filters were used to remove any outlier cells. We removed all cells with < 500 or > 5,000 unique features as well as cells with > 25% mitochondrial RNA. After doublet removal, cells were rescaled and RunPCA was redone. Hypergeometric enrichment analysis was performed using the phyper function (*P* < 0.001). All samples were combined into a single data set, adding metadata with the original sample information. An elbow plot was used to determine the number of principal components to include. Cells were clustered using FindNeighbors and FindClusters and were visualized by RunUMAP. The combined data set was used for downstream analyses including finding biomarkers and differential expression comparisons between the clusters. Differential expression analysis was performed using FindAllMarkers (min.pct = 0.25, log_2_FC > 1).

For the GEO data set GSE165784, we downloaded the raw data file deposited on GEO and then converted the Gexscope to Cell Ranger output file. The matrix files and feature files summarizing the alignment results were analyzed in Seurat (Satija Lab, NYGC) for further analysis. Each sample underwent quality control measures as were performed for our 4 samples.

### Pathway enrichment analysis.

The pathway enrichment analysis was performed using the g:GOSt functional profiling tool available on the g:Profiler web server (version e99_eg46_p14_f929183) to identify significant pathways that distinguish the clusters. Gene Ontology (GO), Kyoto Encyclopedia of Genes and Genomes (KEGG), and Reactome (REAC) databases were used as sources. The enrichment data were used to generate the dot plots in R using *PathFindR* package. Only the most significant differentially upregulated and downregulated genes (log_2_FC > 1, logFC < –1 and *P*_adj_ < 0.01) were chosen for pathway enrichment analysis.

### Cell inference trajectory analysis.

A trajectory analysis was performed using Monocle3 workflow (https://cole-trapnell-lab.github.io/monocle3/docs/trajectories/; https://github.com/katiacoranoscheri/PDR-trajectory-analysis) (59–62). The Seurat object was imported into Monocle data set using the *Monocle3* conversion tool “as. cell_data_set ()” function. After calculating size factors and estimating dispersions, differentially expressed genes among clusters along the trajectory were identified via the “differentialGeneTest.” Monocle tools were used to build the graph and to order the cells. A UMAP was then generated illustrating the trajectory across the clusters.

### CellPhoneDB analysis.

Differential gene expression was performed among stromal cells, endothelial cells, and inflammatory cells in the data set using the Seurat tool FindallMarkers. Cell barcode and counts were extracted from the Seurat object and used in conjunction with the statistical_analysis module of CellphoneDB to generate mean expression values and *P* values. The dot_plot module of CellphoneDB was then used, with the statistical analysis results, to generate dot plots of the pathways.

### Cell culture.

HRP cells (ACBRI 183, Cell Systems) were cultured at 37°C, 5% CO_2_, in Complete Classic Medium with Serum and Culture Boost (4Z0-500, Cell Systems).

For in vitro studies these cells were treated with 10 ng/mL human TGF-β (R&D**)**, cultured in 30 nM D-Glucose or 24.6 nM Mannitol for 24 hours and 48 hours. After the time course, the RNA was extracted for molecular analyses.

### siRNA transfection.

HRP were transfected with 50 nM of siRNA against *AEBP1* (J-009694-05- 0005, Horizon) or 50 nM of a nontargeting siRNA (D-001810-01-05, Horizon) in antibiotic-free complete medium. Dharmafect 2 (T-2002-03, Dharmacon, Horizon) was used for transfection at 0.5 μL/100 μL medium following the manufacturer’s instructions. Twenty-four hours after transfection, medium was changed to serum-free, antibiotic-containing medium, and cells were cultured in either normal-glucose or high-glucose medium for 24 hours as described above. Cells were then lysed for RNA extraction and molecular analyses or fixed in 4% PFA for the immunofluorescence. Target mRNA sequences for siRNAs used: *AEBP1* – GGUGGUGGCUCGUUUCAUC, nontargeting – UGGUUUACAUGUCGACUAA.

### cDNA synthesis and qPCR.

cDNA was made with a SuperScript III First-Strand kit following manufacturer instructions (18080051, Invitrogen). A total of 2 ng of cDNA was used as a template for qPCR, which was performed using PowerUp SYBR Green Master Mix (A25742, Applied Biosystems) following manufacturer’s instructions. qPCR was performed on a QuantStudio3 system (Applied Biosystems). Thermal cycling conditions consisted of 50°C for 2 minutes, followed by an initial denaturation step at 95°C for 10 minutes and then 40 cycles of 95°C 15 seconds and 60°C for 1 minute. A melt curve stage was used for quality control assessments. Relative gene expression between samples was used following standard 2^–ΔΔCt^ methods. See Table 1 for all human primer sequences.

### Immunofluorescence and confocal microscopy.

PDR membranes and the HRP were fixed in 4% PFA, and antigens were retrieved with Antigen Retrieval Solution (0.1M Sodium citrate buffer, pH 6) for 20 minutes at 90°C. Nonspecific bindings were blocked with 1× PBS/1%BSA/0.1% TritonX-100/5% donkey serum for 1 hour at room temperature (RT). The membranes were then incubated with primary antibodies overnight at 4°C, followed by incubation with secondary antibodies for 1 hour and 30 minutes at RT (see Table 2 for primary and secondary antibodies). The membranes were counterstained with DAPI (D1306, Thermo Fisher Scientific) before being mounted with ProLong Gold Antifade reagent (P36930, Thermo Fisher Scientific).

Images of the membrane were acquired with Nikon W1 Dual cam spinning disk confocal laser microscope system (Nikon, Tokyo, Japan) at the Center for Advanced Microscopy/Nikon Imaging Center of Northwestern University.

The corrected total cell fluorescence (CTCF) for AEBP1 was quantified using imageJ software (NIH).

Negative control images with rabbit, goat, sheep, or mouse IgG and secondary antibodies are shown in Supplemental Figure 8.

### Statistics.

Statistical analyses were performed using GraphPad Prism version 9 software (GraphPad Software) for 2-tailed Mann-Whitney *U* test or Kruskal-Wallis 1-way ANOVA followed by post hoc analysis, or using 2-way ANOVA. The data are reported as mean ± SEM. Statistical significance was defined as *P* < 0.05.

### Study approval.

Patients with PDR who presented to the retina clinic with complications that required pars plana vitrectomy (PPV) were recruited from February 2019 to November 2022. The IRB of Northwestern University reviewed and approved the study design and deemed this study minimal risk, not requiring an informed consent. All the procedures were in accordance with Declaration of Helsinki for research involving patients.

### Data availability.

scRNA-Seq data are uploaded to NCBI GEO database under accession no. GSE245561. The Supporting Data Values file, including values for all data points shown in graphs, is available in the supplement.

## Author contributions

Conceptualization was contributed by KCS, JAL, and AAF. Investigation was contributed by KCS and TT. Formal analysis was contributed by KCS, JAL, and BRT. Writing of the original draft was contributed by KCS. Review and editing of the manuscript was contributed by AAF, JAL, and BRT. Visualization was contributed by KCS, JAL, and AAF. Supervision was contributed by AAF. Funding acquisition was contributed by AAF.

## Supplementary Material

Supplemental data

Supporting data values

## Figures and Tables

**Table 2:**
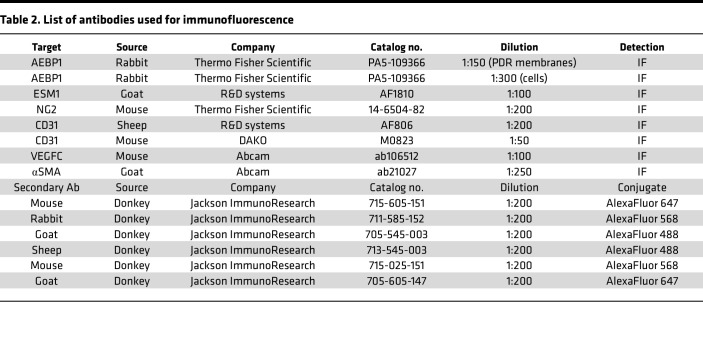
List of antibodies used for immunofluorescence

**Table 1:**
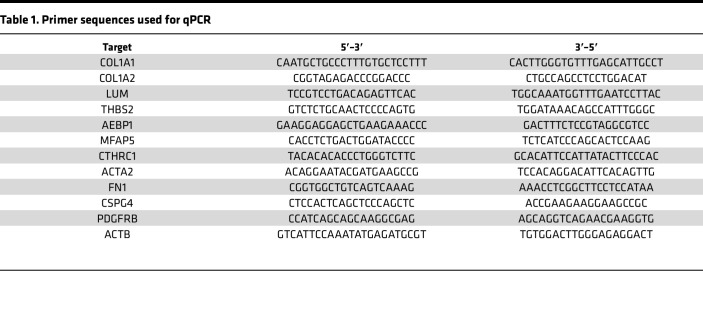
Primer sequences used for qPCR

## Version 1 11/02/2023

In-Press Preview

## Version 2 12/08/2023

Electronic publication
