# Supplementary material for: Single-cell transcriptomics analysis of proliferative diabetic retinopathy fibrovascular membranes reveals AEBP1 as fibrogenesis modulator
Source: JCI Insight. 2023 Nov 2;8(23):e172062. doi: 10.1172/jci.insight.172062 (PMC10896003; doi:10.1172/jci.insight.172062)
Supplement: Supplemental data [file jciinsight-8-172062-s063.pdf]

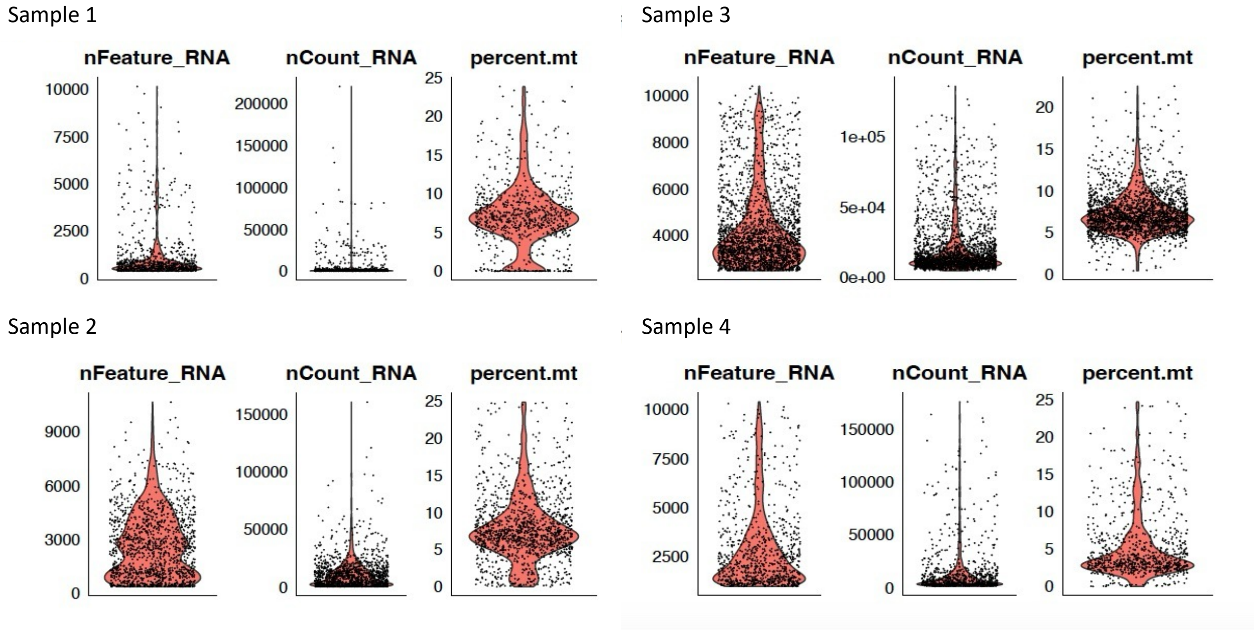

orig.Ident	DoubletClass	N
Sample 1	singlet	581
Sample 1	doublet	5
Sample 2	singlet	1052
Sample 2	doublet	13
Sample 3	singlet	1695
Sample 3	doublet	29
Sample 4	singlet	716
Sample 4	doublet	6

Supplemental Figure 1. Quality controls of scRNASeq analyses

Basic quality check for all the samples submitted to scRNASeq are shown. The violin plots show the nFeature_RNA, nCount_RNA and percent.mt of all the samples. A table showing the singlet and doublet (removed) in each sample is shown.

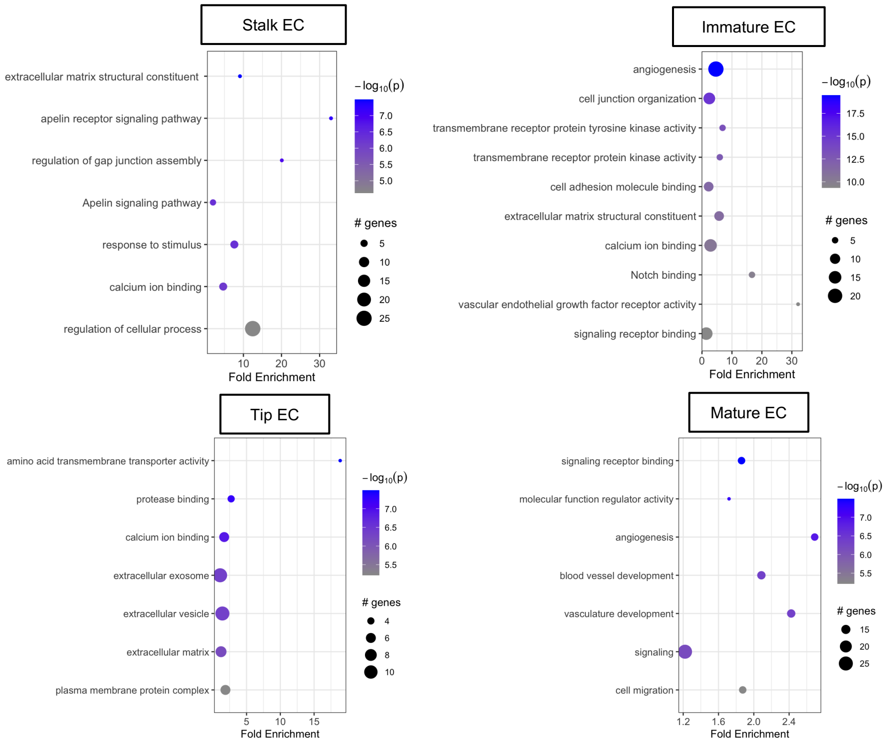

Supplemental Figure 2. Endothelial cell pathway enrichment analysis for the downregulated genes.

Dotplot of the pathway enrichment analysis for the downregulated genes of each cluster using gProfiler and PathFindR package is shown. Only the most significant differentially downregulated genes (log2FC < -1 and p-adj < 0.01) were chosen for pathway enrichment analysis. The graph shows the number of genes modulated in each single pathway, the fold enrichment and the statistical significance.

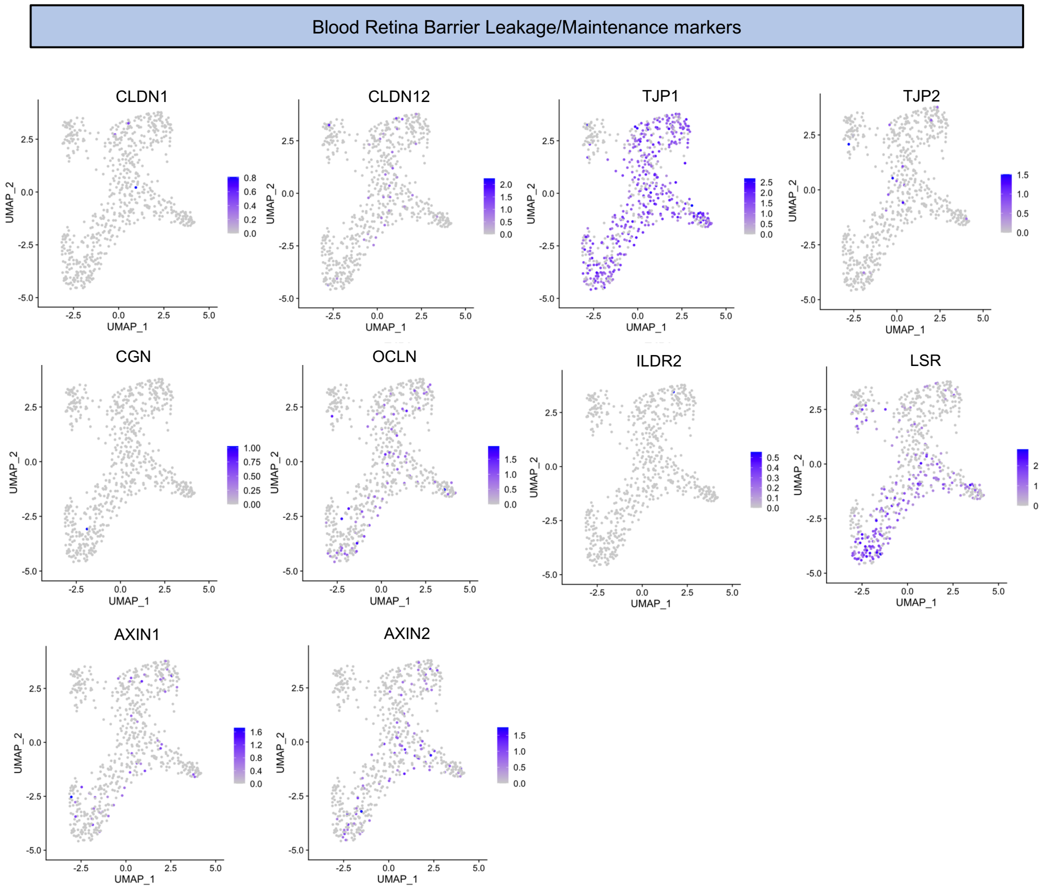

Supplemental Figure 3. Additional blood retina barrier (BRB) markers.

FeaturePlot of additional BRB markers, such as *CLDN1, CLDN12, TJP1, TJP2, CGN, OCLN,* the angulin family genes *ILSR, ILDR2,* and β-catenin target *AXIN1 and AXIN2,* are shown in the panel.

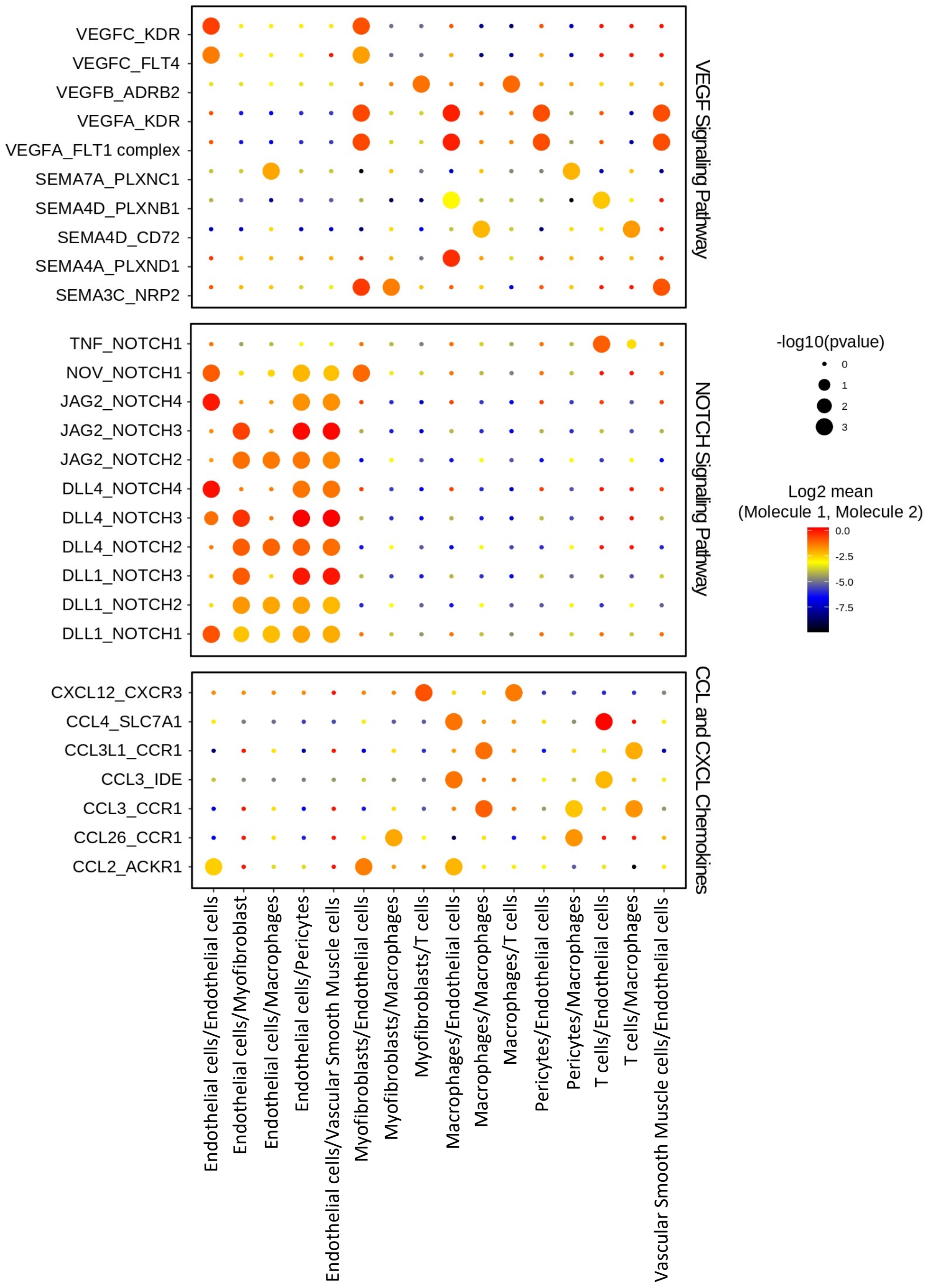

Supplemental Figure 4. Ligand-receptor analyses between endothelial, immune and stromal cells.

Dotplot showing the ligand-receptor interaction of VEGF pathways, Notch pathway and CCL chemokine pathway between endothelial, stromal and immune cells are shown. The first labeled cell type at the bottom corresponds to the first expressed molecule on the left side of the panel.

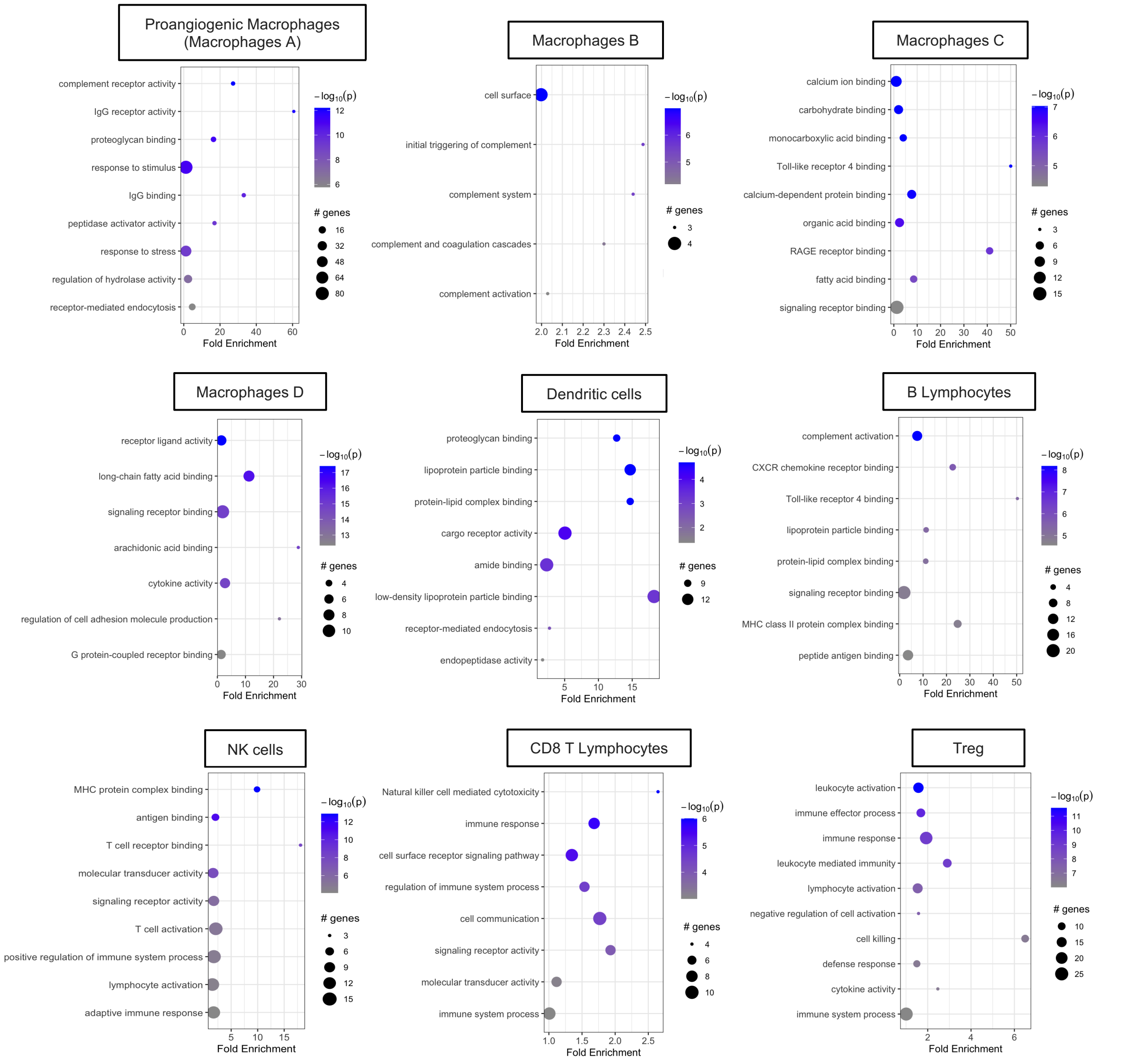

Supplemental Figure 5. Immune cell pathway enrichment analysis for the downregulated genes.

Dotplot of the pathway enrichment analysis for the downregulated genes of each cluster using gProfiler and PathFindR package is shown. Only the most significant differentially downregulated genes (log2FC < -1 and p-adj < 0.01) were chosen for pathway enrichment analysis. The graph shows the number of genes modulated in each single pathway, the fold enrichment and the statistical significance.

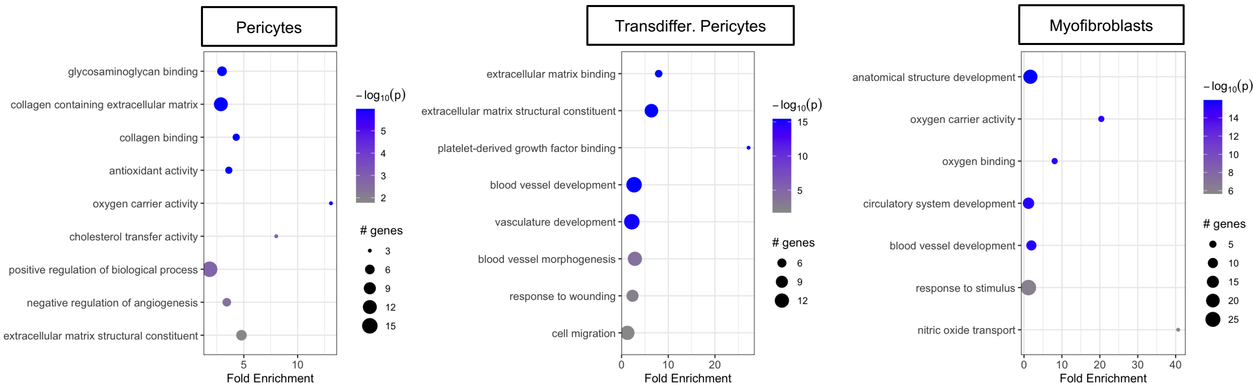

Supplemental Figure 6. Stromal cell pathway enrichment analysis for the downregulated genes.

Dotplot of the pathway enrichment analysis for the downregulated genes of each cluster using gProfiler and PathFindR package is shown. Only the most significant differentially downregulated genes (log2FC < -1 and p-adj < 0.01) were chosen for pathway enrichment analysis. The graph shows the number of genes modulated in each single pathway, the fold enrichment and the statistical significance.

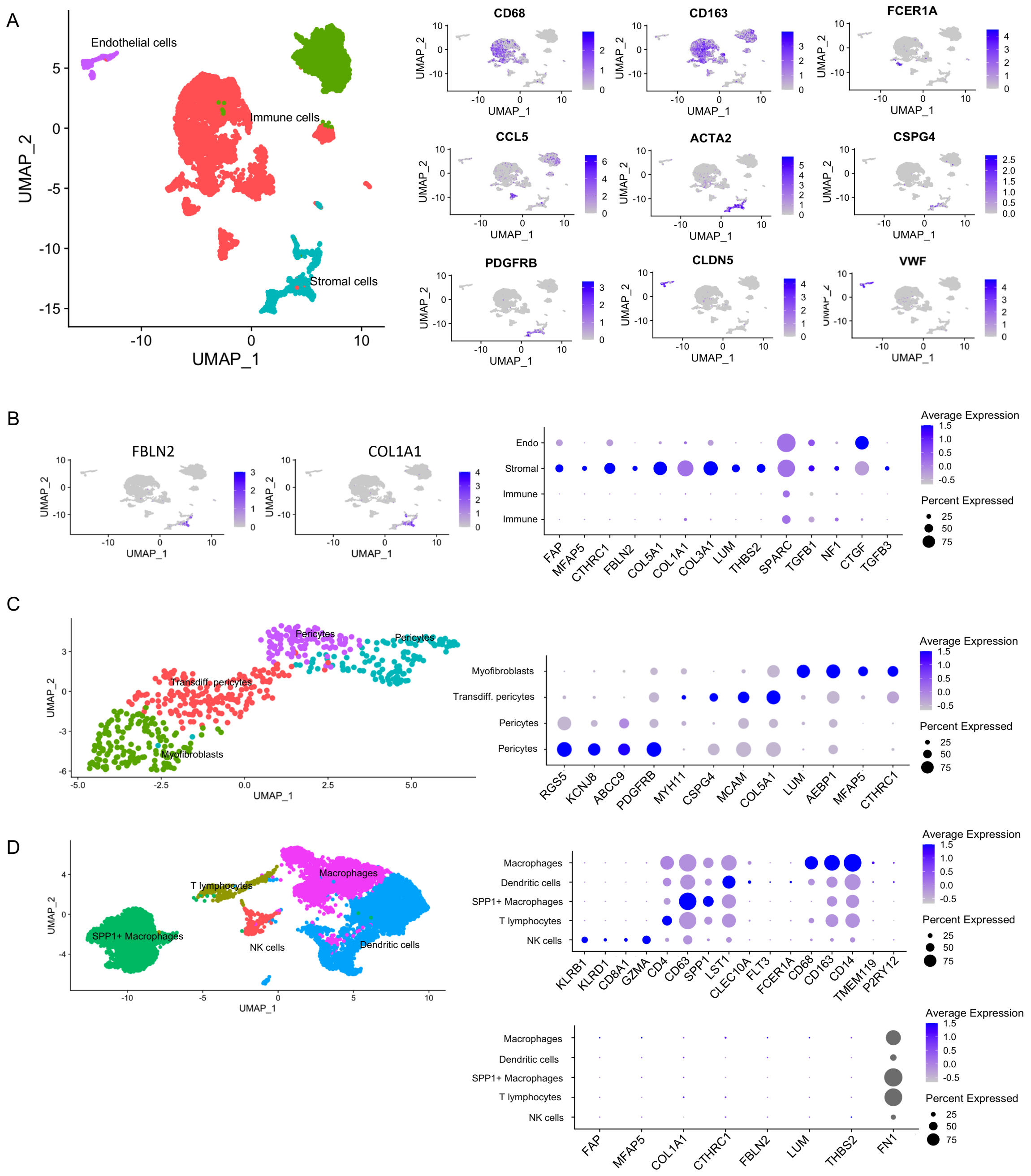

Supplemental Figure 7. scRNASeq analysis of GEO dataset GSE165784.

Representative Uniform Manifold Approximation and Projection (UMAP) plot of the four clusters revealed by Seurat analysis conducted in R Studio, and Feature Plots for cluster identification are shown in A. FeaturePlots and Dotplot for fibrotic markers in all the cell clusters are shown in B. Stromal cell reclustering UMAP and the DotPlot for cell identification are shown in C. Immune cell reclustering UMAP and the DotPlot for cell identification and fibrogenic markers are shown in D.

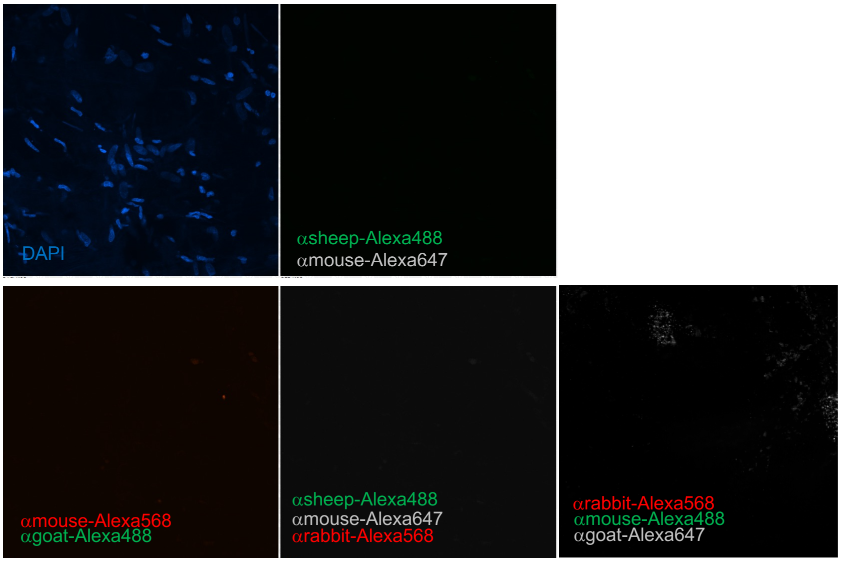

Supplemental Figure 8. Negative control staining

Negative control staining for CD31, ESM1, VEGFC, AEBP1, αSMA and NG2 are shown. Rabbit, goat, sheep or mouse IgG and secondary antibody only were used as negative controls.

Supplemental Table 1. Detailed clinical information of proliferative diabetic retinopathy patients that provided the fibrovascular membranes for single cell RNA Sequencing

Sample #	Age/Sex/Race	DM Type/ Duration (years)/ latest preop A1C %	Kidney failure	Other co-morbidities	RD/ Duration (weeks)	Preop Anti-VEGF/ interval (months)	Laser panretinal photocoagulation/ interval (years)
1	52y/M/B	2/15/12	N	Htn	Y/2	0	Y/2
2	43y/M/H	2/20/9	N	Uveitis	N	0	Y/2
3	29y/F/B	1/18/6	N	Htn	N	1/7	Y/0.3
4	32y/F/H	1/23/9	N	Htn	Y/4	1/27	Y/2

M=male; F=female; B=black; H=Hispanic; DM = Diabetes Mellitus; Preop = Preoperative; A1C %= glycated hemoglobin percentage; Htn=hypertension; RD= retinal detachment; Y=yes; N=No
